# Crude oil exposures reveal roles for intracellular calcium cycling in haddock craniofacial and cardiac development

**DOI:** 10.1038/srep31058

**Published:** 2016-08-10

**Authors:** Elin Sørhus, John P. Incardona, Ørjan Karlsen, Tiffany Linbo, Lisbet Sørensen, Trond Nordtug, Terje van der Meeren, Anders Thorsen, Maja Thorbjørnsen, Sissel Jentoft, Rolf B. Edvardsen, Sonnich Meier

**Affiliations:** 1 Institute of Marine Research, P.O. Box 1870, Nordnes, NO-5817, Bergen, Norway; 2 Centre for Ecological and Evolutionary Synthesis (CEES), University of Oslo, P.O. Box 1066, Blindern, NO-0316 Oslo, Norway; 3 Northwest Fisheries Science Center (NOAA), 2725 Montlake Blvd. East, Seattle, WA 98112-2097, USA; 4 Institute of Marine Research (IMR), Austevoll Research Station, and Hjort Centre for Marine Ecosystem Dynamics, NO-5392 Storebø, Norway; 5 University of Bergen, P.O. Box 7800, NO-5020 Bergen, Norway; 6 SINTEF Materials and Chemistry, P.O. Box 4760, Sluppen, NO-7465 Trondheim, Norway; 7 Department of Natural Sciences, University of Agder, NO-4604 Kristiansand, Norway

## Abstract

Recent studies have shown that crude oil exposure affects cardiac development in fish by disrupting excitation-contraction (EC) coupling. We previously found that eggs of Atlantic haddock (*Melanogrammus aeglefinus*) bind dispersed oil droplets, potentially leading to more profound toxic effects from uptake of polycyclic aromatic hydrocarbons (PAHs). Using lower concentrations of dispersed crude oil (0.7–7 μg/L ∑PAH), here we exposed a broader range of developmental stages over both short and prolonged durations. We quantified effects on cardiac function and morphogenesis, characterized novel craniofacial defects, and examined the expression of genes encoding potential targets underlying cardiac and craniofacial defects. Because of oil droplet binding, a 24-hr exposure was sufficient to create severe cardiac and craniofacial abnormalities. The specific nature of the craniofacial abnormalities suggests that crude oil may target common craniofacial and cardiac precursor cells either directly or indirectly by affecting ion channels and intracellular calcium in particular. Furthermore, down-regulation of genes encoding specific components of the EC coupling machinery suggests that crude oil disrupts excitation-transcription coupling or normal feedback regulation of ion channels blocked by PAHs. These data support a unifying hypothesis whereby depletion of intracellular calcium pools by crude oil-derived PAHs disrupts several pathways critical for organogenesis in fish.

Oil spills in critical fish spawning habitats or aquaculture areas are a recurrent worldwide problem^1^,^2^,^3^,^4^. Expansion of oil production in Northern Norway has been proposed for the Lofoten and Vesterålen regions, which are also crucial spawning and nursing grounds for three of the most important commercial fish species in the North Atlantic, including Atlantic haddock (*Melanogrammus aeglefinus*), cod (*Gadus morhua*) and herring (*Clupea harengus*)^5^. Consequently, there is considerable political and public debate with respect to adverse impacts on these areas from either accidental oil spills or chronic exposure to operational discharges from oil platforms^5^,^6^,^7^,^8^,^9^. A baseline understanding of crude oil toxicity to these species is essential for regulatory decision making, spill response planning, and environmental risk assessment of oil production impacts in spawning and nursery habitats. We recently identified a unique susceptibility of haddock embryos to toxicity from dispersed crude oil^10^. Unlike other species studied to date, the haddock eggshell (chorion) binds oil droplets, enhancing exposure to toxic compounds within crude oil.

Crude oil is a complex chemical mixture containing hundreds of thousands of different compounds. However, polycyclic aromatic hydrocarbons (PAHs) and their alkylated homologues are believed to be the main bioavailable components that are toxic to developing fish^11^,^12^. Multiple lines of evidence accumulated over the last two decades indicate that tricyclic subclasses of PAHs disrupt cardiac function, leading to developmental defects in the hearts of exposed embryos^11^,^13^,^14^. There are several mechanisms by which these PAHs may impact heart development. First, some PAHs activate the aryl hydrocarbon receptor (AhR), which is central to the metabolic detoxification of PAHs as the primary transcriptional activator for PAH–degrading enzymes such as cytochrome P4501A (Cyp1a). Despite this protective role, activation of AhR in the developing heart by some PAHs^15^,^16^ and other potent agonists such as dioxins and PCBs disrupts normal cardiac morphogenesis^17^,^18^,^19^. In these cases, activation of AhR2 specifically in cardiomyocytes is necessary and sufficient for induction of all toxicity phenotypes, including secondary responses such as jaw malformation^18^. However, water soluble fractions of crude oil typically lack PAHs that cause AhR-dependent cardiotoxicity, such as benzo(a)pyrene, and induce heart deformities through a second mechanism that is both AhR-independent^20^,^21^,^22^ and for which Cyp1a metabolism is entirely protective^23^. Studies encompassing crude oils from several sources, individual PAHs, and multiple fish species indicate a common cardiotoxic response in exposed embryos that includes defects in contractility and heart rate and rhythm^13^,^14^,^20^,^21^,^22^,^24^,^25^,^26^,^27^. In particular, these and other studies^11^,^28^,^29^ have linked this cardiotoxicity of crude oil to tricyclic families of PAHs, independent of crude oil source. Single compound studies showed that representatives of the most abundant tricyclic PAHs in crude oils mimic human pharmaceuticals with well known ion channel blocking activity, such as terfenadine^30^, consequently producing a chemical phenocopy of mutations in key ion channels regulating heart rate and rhythm^13^,^20^,^30^,^31^,^32^.

The cellular basis for serious whole-heart arrhythmia caused by drugs such as terfenadine is the disruption of excitation-contraction (EC) coupling^33^, the process that links electrical excitation of cardiac muscle cells (cardiomyocytes) to contraction^34^,^35^. Ca^2+^ and K^+^ ions play central roles in control of the cardiac contraction cycle. Membrane depolarization by action potentials opens L-type Ca^2+^ channels (Ltcc), resulting in extracellular Ca^2+^ entry and a subsequent internal release of Ca^2+^ from the sarcoplasmic reticulum through the ryanodine receptor (RyR). Elevated intracellular Ca^2+^ activates contractile actin/myosin filaments, causing cardiomyocyte contraction. Baseline levels of intracellular Ca^2+^ and K^+^ ions are then restored by the action of pumps and exchangers such as sarcoplasmic-endoplasmic reticulum calcium ATPase 2 (Serca2) and sodium/calcium exchanger 1 (Ncx1), and voltage-gated K^+^ channels such as Erg (encoded by *kcnh2*)^34^,^35^. Compounds that block Erg (e.g., terfenadine) prolong action potentials, leading to reduced heart rate (bradycardia) and arrhythmia^30^, while disruption of Ca^2+^ cycling leads to reduced contractility^36^. Both of these effects were observed in isolated fish cardiomyocytes following crude oil exposure in a manner that was dependent on the concentration of tricyclic PAHs^24^. Therefore crude oil exposure leads to blockade of both Erg channels and intracellular Ca^2+^ cycling mediated by RyR or Serca2^24^, thus providing an AhR-independent mechanism explaining the whole heart effects observed in exposed embryos (e.g. refs 14 and 27).

Ion channels are typically composed of complex heteromultimeric proteins. The role for gene transcription in homeostatic regulation of channel biosynthesis is not well characterized^37^. Ion channel homeostasis theoretically involves both cognate (self) regulation of an affected channel at multiple levels (e.g., transcriptional, post-transcriptional, and multimer assembly), or compensatory regulation through other ion channels functioning in the same pathway (e.g., EC coupling)^37^. However, pharmacological blockers of voltage-gated sodium channels and pacemaker channels have been shown to increase the expression of the cognate channel genes^38^,^39^. Thus, a transcriptional response might be expected for genes encoding those EC- coupling channels that are blocked by oil exposure.

The developing fish heart acquires functional pumping activity very early during its complex morphogenesis. Because cardiac form and function are tightly intertwined, defects in cardiac function at early developmental stages can profoundly influence subsequent morphogenetic steps^40^. The fish heart develops from a simple tube that is segmented into two chambers, the atrium and ventricle, which subsequently “loop” to adopt a side-by-side orientation. The early contractile heart tube is formed from mesodermally-derived cardiomyocytes arising from the first heart field. As the heart tube expands, cardiomyocytes and cells contributing to the outflow tract are added from two other sources, the mesodermal second heart field and neural crest cells that migrate from the neural tube^41^,^42^. In zebrafish (*Danio rerio*), abnormal looping and ventricular development are associated with genetic defects in both EC coupling and contractile function. For example, normal intracellular Ca^2+^ levels are necessary for cardiomyocyte proliferation^36^,^43^, while reduced atrial contractility leads to altered ventricular morphology^44^. Finally, activation of AhR in ventricular cardiomyocytes reduces their proliferation^18^,^45^. Therefore, there are several routes by which the molecular targets of crude oil can impinge upon cardiac morphogenesis.

We previously demonstrated that Atlantic haddock embryos were unusually susceptible to relatively low levels of dispersed crude oil (3 μg/L ∑PAH), due in part to oil droplet binding to the eggshell^10^. However, in that study, the animals were exposed relatively late in development, after organogenesis and the major steps of heart formation (e.g., looping). The severe effects observed with this late exposure suggest that earlier exposure would have even more profound consequences. Moreover, for purposes of risk assessment, toxicity information is needed for a broader range of developmental time points. To address these data gaps we exposed haddock to very low concentrations of dispersed crude oil (60–600 μg oil/l = 0.7–7 μg/L ∑PAH). Continuous exposure began shortly after fertilization and ended 3 days before hatch. We also conducted transient 24- and 48-hr exposures over the third and fourth days of embryogenesis, prior to development of a functional heart. As in our previous study, we also included an intermittent or pulsed exposure, because it is possible that in an actual spill fish eggs and larvae might experience short-term repetitive exposure as result of transport in and out of high oil concentration areas. Lastly, we exposed larvae from shortly after hatch to 18 days post-hatch. For both embryos and larvae, we quantified effects on cardiac function and morphogenesis. The visible injury phenotype was then compared to the expression of genes encoding potential molecular targets of PAHs in EC coupling to further characterize potential mechanisms of crude oil cardiotoxicity and novel craniofacial abnormalities.

## Results

### Eggshell oil droplet accumulation and differential PAH uptake in embryos and larvae

Total PAHs (dissolved and droplet-associated) were measured in water at the start of exposure. For the low and high dose treatments, ∑PAH was 0.58 ± 0.05 μg/L and 6.7 ± 0.2 μg/L, respectively. The pulse dose peak concentration was the same as high, as expected, at 6.8 ± 1.0 μg/L measured at the maximum of the 2.4 hour pulse, and returned to background levels (0.09 ± 0.02 μg/L) between pulses. ∑PAH concentration in the control tanks was 0.03 ± 0.01 μg/L. For the larval exposure, low and high dose treatments had 0.65 ± 0.08 μg/L and 7.6 ± 0.7 μg/L, respectively. The pulse exposure had a peak concentration of 6.1 ± 0.5 μg/L, with a low concentration of 0.3 ± 0.3 μg/L between pulses. Control tanks in the larval exposure had ∑PAH concentrations of 0.03 ± 0.01 μg/L. The PAH profile in the exposure tanks was dominated by the C0-C3 naphthalenes (~60%), followed by the tricyclic PAHs which contributed ~39% of the total PAHs (Supplementary Fig. S1, Supplementary Table S1).

**Figure 1:**
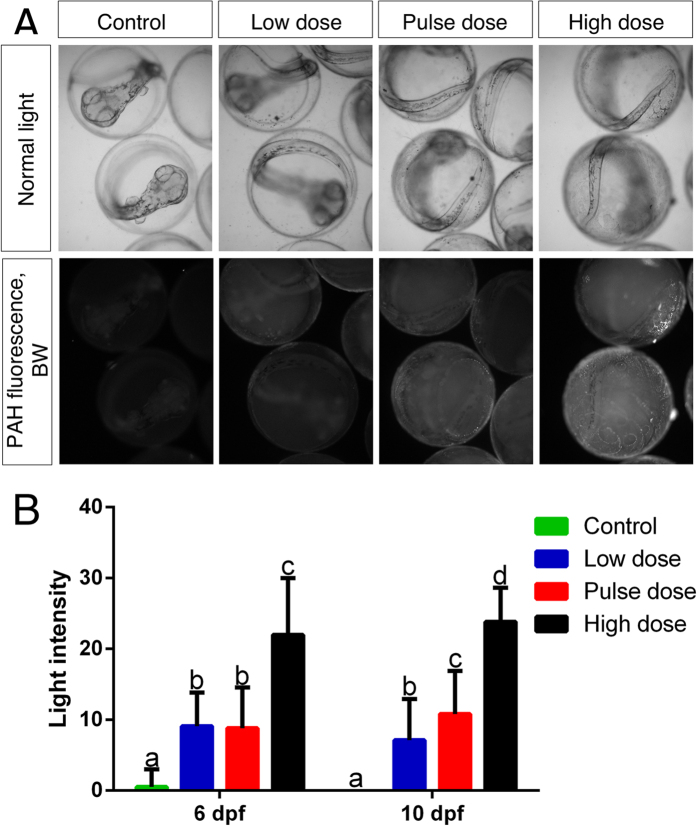
PAH-associated fluorescence. (**A**) 10 dpf embryos (last day of exposure) in transmitted light (first panel) and PAH-associated fluorescence as black and white (second panel). (**B**) measured light intensity in control, low, pulse and high dose at 6 dpf and 10 dpf. Statistical significant difference between groups (p < 0.05) is indicated with letters, (i.e., groups with same letters are not significantly different from each other).

The adherence of oil microdroplets to the eggshell was evident microscopically within 24 hours of embryonic exposure, and this increased over time (Supplementary Fig. S2). Fluorescence from oil-associated compounds such as PAHs showed that by 10 dpf droplets coalesced nearly into a sheet covering the eggshell (Fig. 1A) in a concentration-dependent manner (Fig. 1B).

**Figure 2:**
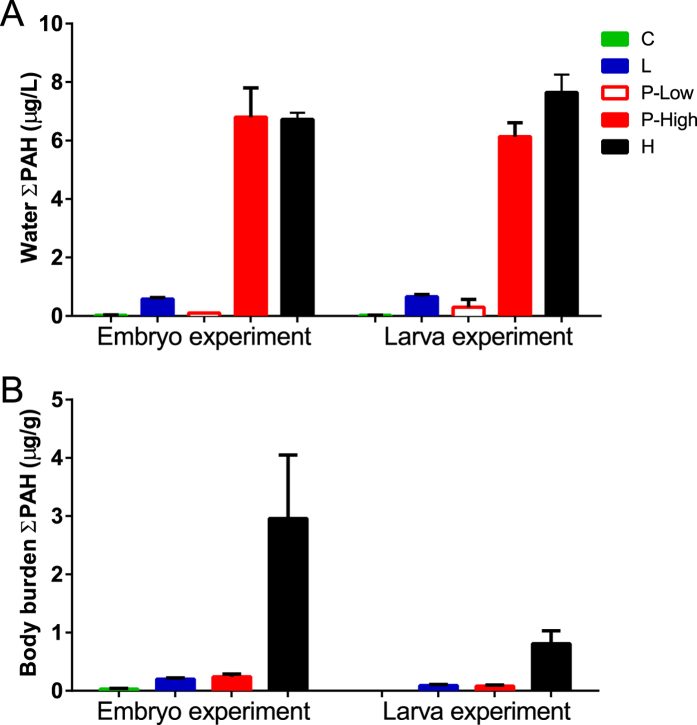
Total measured PAH. (**A**) Water concentrations of ∑PAH (ug/L) and (**B**) body burden of ∑PAH in embryo and larval exposure at the end of the exposure (10 dpf and 18 dph, respectively).

Tissue concentrations of PAHs in embryos and larvae were determined at the end of each exposure. These were higher for all treatments with embryos relative to those with larvae (Fig. 2). Embryos accumulated ∑PAH concentrations (ng/g wet weight ± SD) of 27 ± 10, 192 ± 17, 218 ± 64, and 2961 ± 1258 and larvae accumulated 5 ± 1, 86 ± 15, 81 ± 24, 808 ± 184 ng/g ∑PAH in control, low, pulse and high dose treatments, respectively. Although the higher levels of embryo-associated PAHs were evidently due in part to whole oil coating the eggshell, it was not possible to differentiate between this fraction and the PAH fraction that was bioavailable to haddock embryos within these ∑PAH measures. Embryos from one low dose and pulse dose tank (L3 and P1, respectively) had lower ∑PAH concentrations than the other replicates, and both were closer to each other. Embryos in the L3 tank had ∑PAH levels of 226 μg/g compared to 274 ± 15 (mean ± SD) for the other replicates, while embryos from the P1 tank had ∑PAH of 188 μg/g compared to 376 ± 93 for the other pulse treatment replicates. As indicated below and in Supplementary Fig. S3, fish from these two tanks displayed correspondingly reduced toxicity.

### Temporal and tissue expression patterns of *cyp1a* induction in relation to exposure

We measured the effective response to tissue uptake of PAHs using quantitative PCR (qPCR) for *cyp1a* mRNA in pooled samples. Two-way ANOVA showed significant effect of time, dose and time-dose interaction for both embryonic and larval exposure (Table S2). Remarkably, *cyp1a* induction was evident within 24 hours of exposure in the high dose group when embryos were starting gastrulation (~50% epiboly) (p = <0.0001). Expression of *cyp1a* increased during exposure with a plateau at 8 dpf of roughly 50-, 70- and 180-fold in low, pulse and high dose treatments (p < 0.0001), respectively. One day after transfer to clean water, but prior to the onset of hatching (11 dpf), *cyp1a* expression remained at plateau levels in each exposed group (p < 0.0001). However, after initiation of hatching *cyp1a* expression in exposed animals dropped to control levels (Supplementary Fig. S4). In the embryonic exposure, the L3 (low dose) and P1 (pulse dose) replicates showed 5-times lower *cyp1a* expression relative to the other 3 tanks receiving the same dose at all sampling points during exposure, and also showed lower frequencies of toxicity-related effects (see below), consistent with reduced exposure to oil. Although a Grubbs’ test of individual *cyp1a* or ∑PAH data from 10 dpf (final day of exposure) was inconsistent with these points being outliers, plotting *cyp1a* levels against ∑PAH concentrations indicated that these two tanks can be considered a separate, lower dose (Supplementary Fig. S3). The lower biological response during the whole time series indicates that these two replicates received a lower internal dose. This may not be reflected in the measured tissue PAHs, which includes biologically unavailable material on the outer eggshell. Therefore, we excluded data from those tanks in statistical analyses of exposure and effects endpoints below. Compared to haddock exposed as embryos, *cyp1a* was rapidly induced in larvae and maintained at a significantly lower (p < 0.0001) but relatively constant level, roughly 8-, 13- and 18-fold in low, pulse and high dose, respectively (Supplementary Fig. S4).

As a measure of AhR activation in tissues in relation to toxicity phenotypes, we determined the tissue distribution of Cyp1a. In the embryonic exposure, we used whole mount immunofluorescence and confocal microscopy in newly hatched larvae (11 dpf) (Supplementary Fig. S5). Control larvae showed no Cyp1a labeling, while larvae from the low dose group showed low levels of Cyp1a labeling in the epidermis and liver. Larvae from the high dose group showed labeling that was visible throughout the epidermal cells and strong in the liver. Remarkably, there was little if any Cyp1a protein detected in the heart, either in the endothelial layer or myocardium. No Cyp1a labeling was observed in other internal structures besides the liver in exposed larvae.

For the larval exposure, we examined the tissue distribution of *cyp1a* mRNA by *in situ* hybridization at 1 dph and 18 dph. After one day of exposure (1 dph), *cyp1a* expression was evident in the outer epidermis, liver hepatocytes, epithelia of the head kidney and intestines, and endothelium of the heart, but notably absent from the myocardium (Supplementary Fig. S6). After 18 days of exposure (18 dph) *cyp1a* expression was maintained in the epidermis, heart, kidney, intestines and liver, and new expression was observed in the gall bladder, gills and brain. In contrast to 1 dph where *cyp1a* expression was pronounced in hepatocytes, expression appeared to be limited to the sinusoid lining cells at 18 dph (Supplementary Fig. S6). Moreover, at 18 dph, the liver sinusoids of oil exposed larvae appeared more dilated in comparison to control larvae, consistent with our previous observations^10^.

### Stage-specific craniofacial malformations

**Figure 3:**
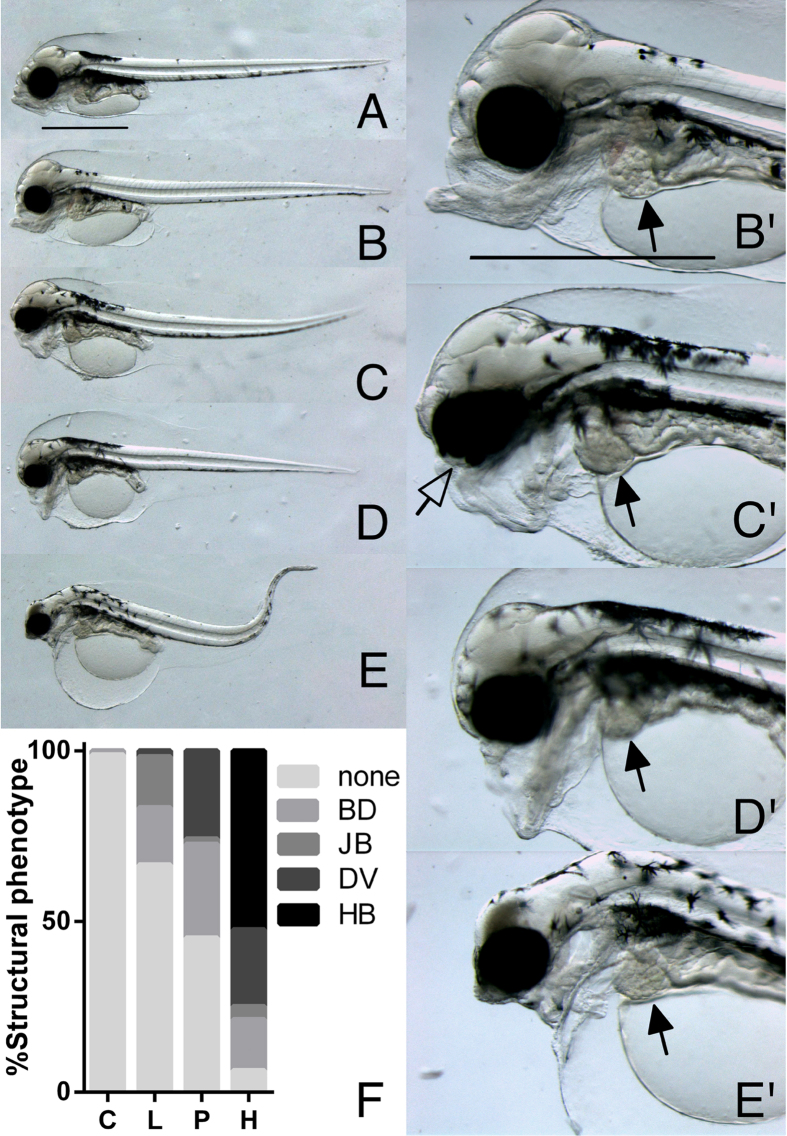
Craniofacial phenotypes following embryonic exposure. (**A**) Control and (**B–E**) exposed animals at 3 dph (6 days post exposure stop). Craniofacial malformations fell into four distinct phenotypes: (**B**,B’, higher magnification) bulldog phenotype, BD, (**C**,C’) jaw breaker, JB (**D**,D’) Darth Vader, DV and (**E**,E’) hunchback, HB. Filled arrows indicate livers and guts appearing to be grossly normal in comparison to craniofacial and cardiac abnormalities. Filled arrow in C’ indicates coloboma. (**F**) Distribution of structural phenotypes with percentage of animals with no abnormal phenotypes = none, BD, JB, DV and HB phenotype for control = C, low = L, pulse = P and high = H doses). Scale bar 1 mm.

Embryonic exposure resulted in a dose-dependent gradient of striking craniofacial malformations visible by 3 dph (Fig. 3). These abnormalities were in addition to the pericardial/yolk sac edema that is characteristic of crude oil cardiotoxicity (detailed below). Exposure to increasing concentrations of oil led to a progressive loss of craniofacial structures, affecting the upper jaw first at the low dose (Fig. 3B) and including the lower jaw in the high exposure group (Fig. 3E). Craniofacial malformations consistently fell into four distinct types: isolated underdevelopment of the upper jaw (bulldog, BD); a more severe underdeveloped upper jaw with a posteriorly displaced lower jaw (jawbreaker, JB); an underdeveloped upper jaw with hanging lower jaw (Darth Vader, DV); and finally, severe reduction of all jaw structures, reduced anterior brain structures and poor dorsal rotation of the head conveying a characteristic hunchback appearance (hunchback, HB). In addition, all animals with the HB phenotype and many animals with the DV phenotype had dorsal curvature of the body axis and reduction of the posterior finfold. The frequencies of the various phenotypes coincided with dose. The most frequent abnormal phenotypes in low dose group were BD and JB (17% and 15%, respectively), the pulse dose was dominated by BD and DV (28% and 27%, respectively), while the high dose group was dominated by HB (54%), although BD and DV also were frequent (15% and 23%, respectively) (Fig. 3F). These phenotypes also occurred after the transient embryo exposure (24 and 48 hours starting at 2 dpf). The 24-hour high dose exposure group had incidences of BD and DV of 7% and 3%, respectively, while the 48-hour high dose exposure group had 17% BD, 10% DV, and even a small percentage of HB (3%) (Table 1).

**Figure 4:**
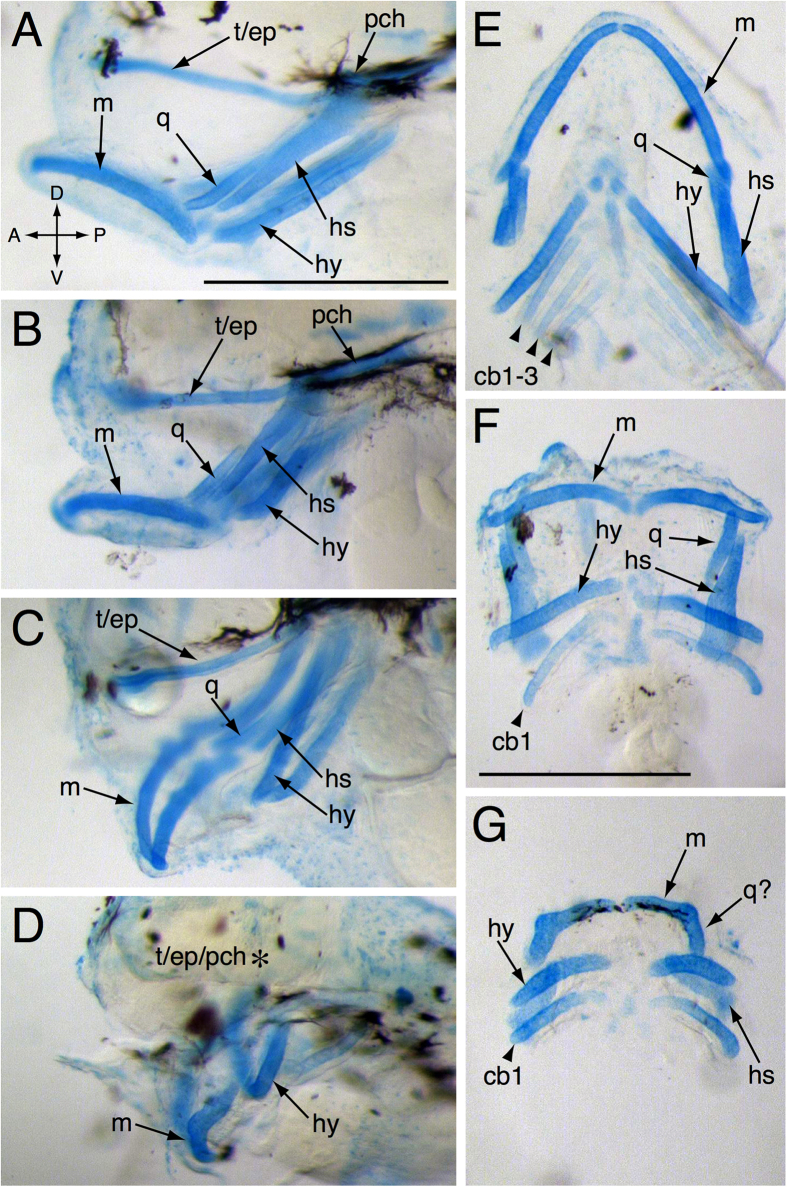
Alcian blue staining of 3 dph embryonically exposed larvae. (**A,E**) show a lateral and dorsal view of a control larva, respectively. (**B–D**) and (**F–G**) show the lateral and dorsal views of exposed larvae, respectively. t/ep = trabeculae crania/ ethmoid plate, pch = parachordal cartilages, m = Meckel’s cartilage, q = quadrate, hs = hyomandibular symplectic pharyngeal arches, hy = hyoid pharyngeal arches, cb = ceratobranchial Asterisk in (D) indicates absent basicranial cartilages Scale bar 0.5 mm.

Alcian blue staining at 3 dph confirmed a graded and overlapping series of defects in the cartilaginous precursors of the craniofacial skeleton (Fig. 4). The head skeleton consists of three basic divisions: the basicranium, the jaws and the gill support structures. Cartilages present at this stage in controls included the basicranium, comprising the ethmoid plate, trabeculae and parachordal cartilages (Fig. 4A,E), and main jaw cartilages derived from the first (Meckel’s and quadrate), and second (hyoid and hyomandibular-symplectic) pharyngeal arches (Fig. 4A,E). While the posterior pharyngeal arches produce five ceratobranchials, only three were visible by Alcian blue staining at this stage (Fig. 4). In the milder “short face” or BD phenotypes all cartilages were present, but they were generally reduced in length (Fig. 4B). After Alcian blue staining, it was difficult to distinguish between DV and JB animals. However, these phenotypes were both characterized by presence of the main jaw cartilages derived from the first and second pharyngeal arches, with distortion of Meckel’s cartilage that varied in severity, but generally was marked by inversion across the anterior-posterior axis (Fig. 4C,F). In addition, ceratobranchials other than cb1 were typically absent (Fig. 4F), and the basicranium often was reduced to a degree that seemed disproportionate to the reductions of the first and second arch derivatives. In the most severe cases (HB), the basicranium was completely lacking, and the first and second arch derivatives were markedly smaller with the quadrate lacking (Fig. 4D,G), and only the first ceratobranchial was present (Fig. 4G). In addition to the anterior-posterior inversion, the misshapen appearance of Meckel’s cartilage suggested that the quadrate may not be simply absent, but fused with Meckel’s cartilage (Fig. 4G).

In addition to jaw malformations, some animals appeared to have more closely spaced eyes (hypotelorism) and a failure to close the choroid fissure (coloboma) (Supplementary Fig. S7). We quantified eye spacing in ventrally positioned larvae at 0 dph. Although all exposed groups showed hypotelorism, this effect was more severe in the low dose group (26% reduction in eye spacing), than both high and pulse groups (14% reduction; Table 1). Because of time limitations related to collection of cardiac function data, we did not collect the types of images necessary for rigorously quantifying the occurrence of coloboma.

Haddock exposed as larvae did not show same the severe phenotypes that were evident following embryonic exposure. After two days of exposure the ethmoid plate was significantly shortened in the high dose group (Supplementary Fig. S8). Shortening of the ethmoid plate was significant and dose-dependent in all groups after 9 days of exposure (9 dph), with lengths of 197-, 199- and 170 μm in low, pulse and high dose, respectively, compared to 219 μm in control (Supplementary Fig. S8 and Table 2). After 18 days of exposure (18 dph) the high dose group showed continued reduction of the ethmoid plate at 48% of control (Table 2). To determine if the ethmoid plate was selectively reduced or simply reflective of a smaller total skull size, we measured the mandible and used the ethmoid plate/mandible (EP/M) ratio. The mandible also was significantly reduced in the high dose group at 2, 9 and 18 dph, in pulse dose group at 9 and 18 dph and in low dose at 2 and 9 dph. However, the EP/M ratio showed that ethmoid plate shortening was greater than shortening of other craniofacial structures in the high dose group at 9 and 18 dph, and in pulse group at 18 dph (Table 2).

### Distinct cardiotoxic effects in embryonic and larval exposures

As expected from previous studies in other species, haddock embryos displayed dose-dependent edema in response to continuous embryonic oil exposure after only 7 days (Table 3). Edema accumulated in both the yolk sac sinus and pericardial space. By hatching stage (13 dpf/0 dph), edema spread more extensively throughout the yolk sac. The percent of area occupied by edema fluid was significantly higher in the low dose group (15%) and the pulse and high dose groups (both 31%) compared to control (7%) (Table 3). Surprisingly, occurrence and severity of edema did not correlate exactly with severity of craniofacial phenotype. For example, exposed animals with either normal or BD craniofacial phenotype sometimes had more edema than animals with JB phenotype. Similarly, larvae with DV phenotype had higher occurrence and more severe edema than those with HB phenotype (Supplementary Fig. S9). In the transient embryo exposure, edema was evident only in embryos exposed to the high dose for 48 hours (17% compared to 10% in controls, Table 3).

Because edema is a sign of reduced cardiac output, we assessed cardiac function with digital video. Bradycardia (reduced heart rate) was observed in animals from the continuous embryo exposure, both during exposure (at 9 dpf) and after exposure (0 dph and 3 dph) (Table 3). In addition some exposed embryos also displayed continuous ventricular asystole (silent ventricle) as early as 9 dpf in a dose-dependent manner, with continuously exposed groups showing 13%, 30%, 54% in low, pulse and high dose, respectively, compared to 5% in control at 0 dph (Table 3). With further development, the fraction of silent ventricles increased so that at 3 dph frequencies were 57%, 29% and 72% in low, pulse and high dose, respectively, compared to 2% in control. Remarkably, animals from both transient exposures also had silent ventricles, with an incidence of 20% in the 24-hour high dose exposure (Supplementary Video S1), and 17%, 7% and 23% in the 48-hour low, pulse and high dose exposures, respectively. In contrast to the continuous exposure, however, transiently exposed embryos did not show bradycardia (Table 3).

As a consequence of the high frequency of silent ventricles, ventricular contractility (fractional shortening, see Methods) at 0 dph was significantly reduced in pulse and high dose, while atrial contractility was solely reduced in the high dose group. By 3 dph the low dose group also had significant occurrence of silent ventricle, thus reducing the contractility measure (Table 3, shown graphically in Supplementary Fig. S10). Because silent ventricles have a fractional shortening measure of zero, this skews the mean measure for the whole group. Elimination of measurements from silent ventricles showed that animals with functional ventricles in low and high dose groups still had significantly reduced ventricular contractility.

**Figure 5:**
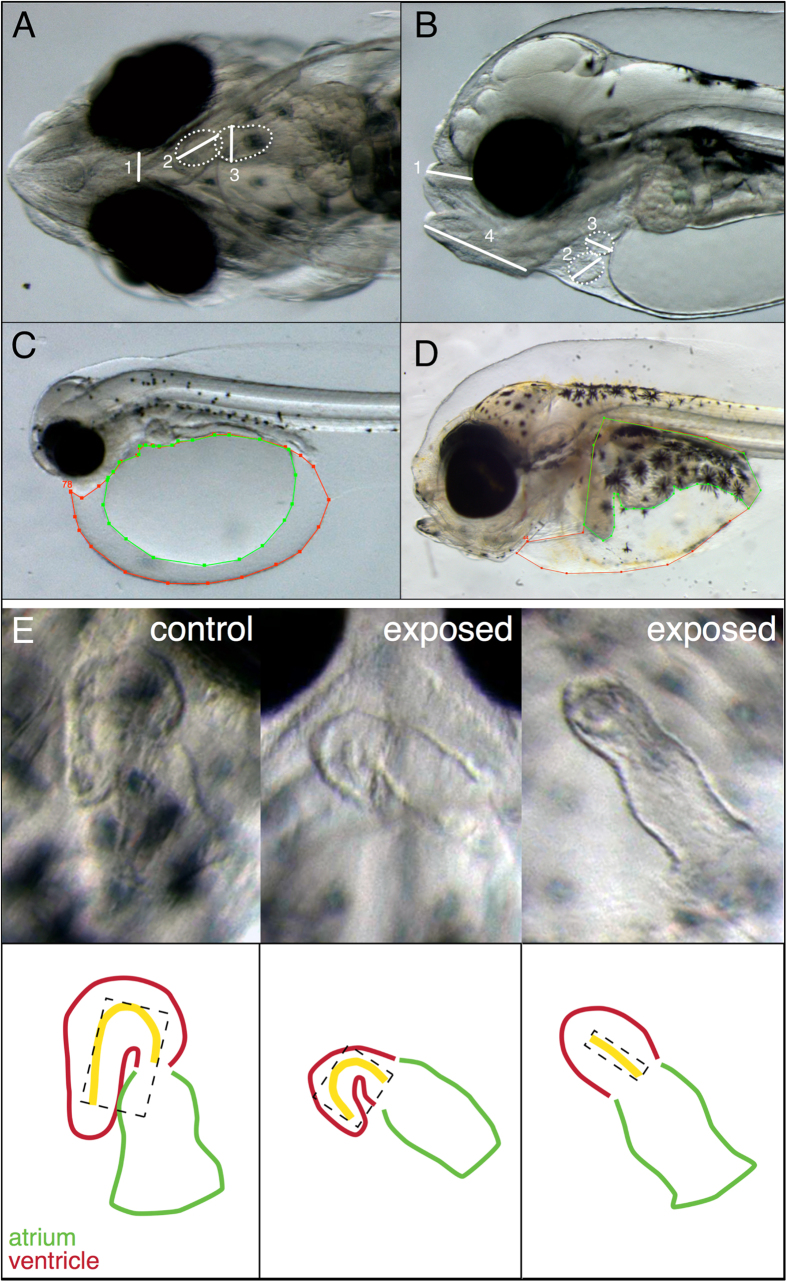
Illustration of larval measurements. (**A**) Measurement of length between eyes (1) and ventricular and atrial diastolic and systolic diameter (2 and 3, respectively) at 0 dph and 2 dph (transient embryonic exposure only), ventrally positioned. (**B**) Measurement of ethmoid plate from eye to nose tip (1), measurement of ventricular and atrial diastolic and systolic diameter (2 and 3, respectively) at 3 dph and 10 dph, laterally positioned and (**C**) measurement of edema at 0 dph and 2 dph (transient embryonic exposure only). Red line: yolk sac area with included edema. Green line: only yolk sac area. (**D**) Edema measurements in 10 dph and 19 dph larvae. Red line represents both organ area and edema. Green line represents only organs. (**E**) Ventricular length (yellow line), area (dashed box) and circularity (yellow line) were measured to estimate cardiac deformity.

Animals with severely reduced cardiac function often had ventricles that appeared markedly underdeveloped. To quantify ventricular form and determine if there were more subtle perturbations in cardiac morphology, we measured the area, length and curvature (as circularity) of the ventricle (Fig. 5E). These measures allowed an assessment of the size and three-dimensional orientation (looping). Animals with HB and DV phenotypes had the most severely impacted cardiac morphology, resulting in a dose-dependent decrease in ventricular length, area and circularity (Table 3). While the low dose group had significantly reduced ventricular area and length, the circularity was not significantly different from control, suggesting an effect on ventricular growth but not looping (Table 3).

**Figure 6:**
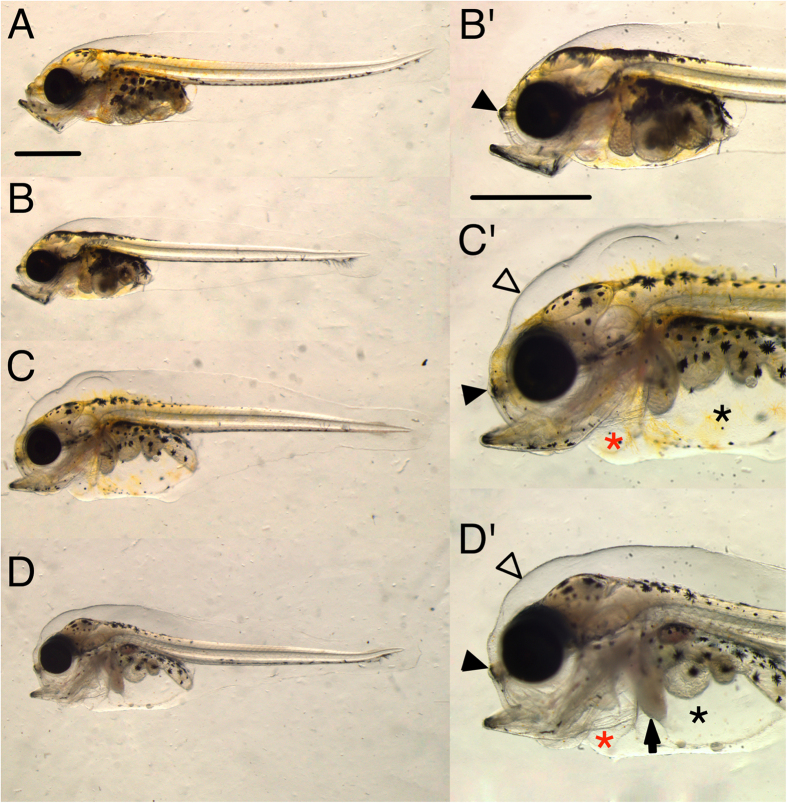
Phenotypes after 18 days of larval exposure. (**A**) Control and (**B–D**) exposed larvae (higher magnification **B**’–**D**’). There was variation in appearance of deformities in all exposed groups, ranging from larvae with shortened ethmoid plate and mild edema in (**B**) to the more extreme cases in (**C,D**) with jaw deformities, severe abdominal (asterisk) and pericardial (red asterisk) edema, shortened ethmoid plate (filled arrowheads), subdermal fluid accumulation (open arrow heads) and opaque liver (arrow). Scale bar 1 mm.

Larval exposure also resulted in edema, but produced different cardiac function phenotypes. Edema accumulated in the pericardial compartment as well as the peritoneal cavity as true ascites, and in the dorsal subdermal space of the head (Fig. 6). After 9 days of larval exposure (9 dph), we observed significantly increased edema area of 12, 12 and 13% in low, pulse and high dose, respectively, compared to 9% in control (Table 3). After 18 days of exposure (18 dph), edema was significantly elevated only in the high dose group (32% compared to 7% in control) (Table 3). In addition to the severe edema found in the high dose larvae at 18 dph, the liver often appeared opaque, an indication of liver toxicity^15^ (Fig. 6).

No changes in larval cardiac function were detected after only 2 days of exposure, but were measureable at 9 days (Table 3). As opposed to the bradycardia observed in embryos, there was a trend towards tachycardia (increased heart rate) (Table 3). We also detected a distinct rhythm abnormality in the form of the heartbeat, bypassing of ventricle, indicative of atrioventricular (AV) conduction block (Table 3, Supplementary Fig. S10, Supplementary Video S2). This ventricular arrhythmia was observed in all exposed groups and was most frequent in the high dose. The arrhythmia ranged from intermittent and random skips of ventricular contraction (asystole) to a regular 2:1 partial AV block (i.e., skipping every other ventricular contraction). Silent ventricle was not observed in the larval exposure. In contrast to the embryonic exposure, ventricular fractional shortening was increased in the high dose group after 9 days of exposure, 21% compared to 17% in control, probably due to increased filling in animals with AV block.

### Relationship of phenotypes to gene expression

**Figure 7:**
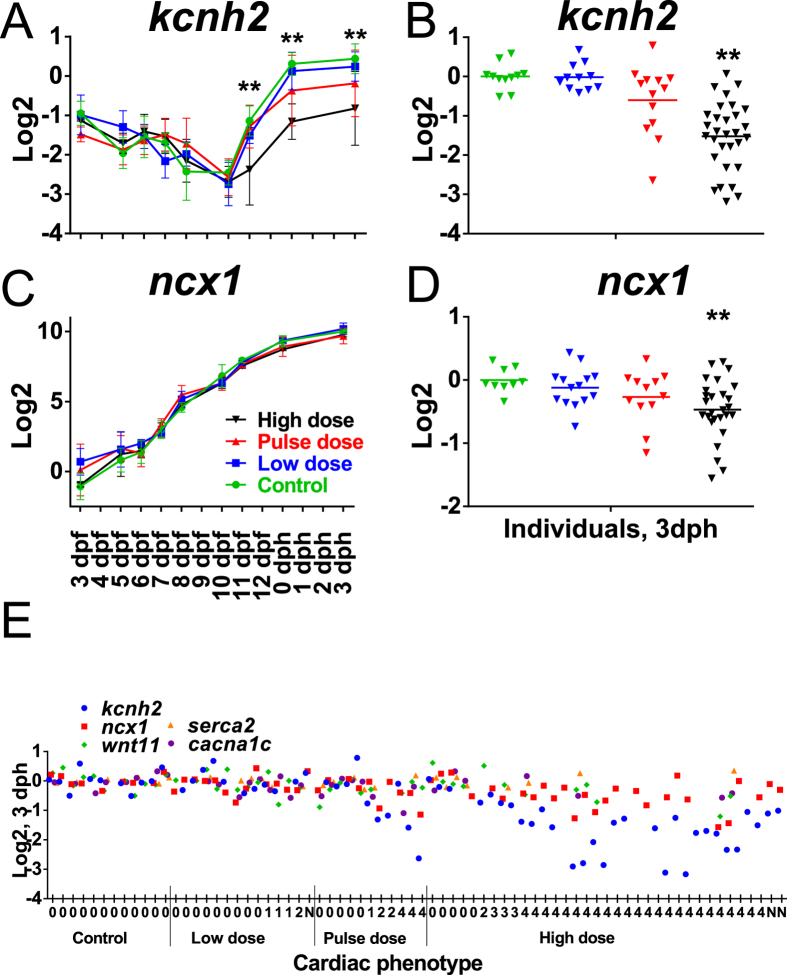
Expression of genes involved in EC coupling and *wnt11* in the embryonic exposure. Expression of *kcnh2* in (**A**) pooled samples throughout early development (3 dpf to 3 dph) and (**B**) individuals at 3 dph (6 days post exposure), and *ncx1* in (**C**) pooled samples and (**D**) individuals. (**E**) Expression of *kcnh2, ncx1, cacna1c, serca2* and *wnt11* in individuals at 3 dph. Collectively, in individuals only *kcnh2* and *ncx1* were significantly different from control. Statistical significant difference from control is indicated by *p < 0.05 and **p < 0.01. Numbers along x-axis refer to cardiac phenotype: 0 = no cardiac abnormalities; 1 = Silent ventricle; 2 = Underdeveloped ventricle; 3 = Silent ventricle and underdeveloped ventricle; 4 = Silent ventricle and very underdeveloped ventricle.

The observed craniofacial and cardiac phenotypes suggest several candidate target genes whose expression level might be perturbed by oil exposure. Based both on the known potassium and calcium channel ion blocking activity of crude oil^24^ and the cardiac function defects observed here, we selected several EC coupling genes, including ncx1 (sodium/calcium exchanger 1 (Ncx1)), cacna1c (voltage-dependent L-type calcium channel (Ltcc)), kcnh2 (potassium voltage-gated channel subfamily H member 2 (Erg)), serca2 (sarcoplasmic-endoplasmic reticulum calcium ATPase (Serca2))^34^ as well as a gene that may be involved in both craniofacial and cardiac defects (wnt11 (wingless-type MMTV integration site family, member 11)^46^,^47^). Gene expression was measured by qPCR in both pools and in individual larvae with known phenotypes. In the embryonic exposure a significant effect of both dose and time and a time-dose interaction were observed only for kcnh2 (Table S2). Dunnet’s post hoc test showed significantly reduced expression in the high dose group at 11 dpf (1 day post-exposure) (p = <0.0001), 0 dph (p = 0.0021) and 3 dph (0.0027) (Fig. 7A). On average, individual larvae showed down-regulation of kcnh2 at 3 dph in accordance with cardiac phenotype (Fig. 7B). Surprisingly, individuals with silent ventricles and relatively normal morphology did not have reduced kcnh2 expression, while those with underdeveloped silent ventricles showed reduced kcnh2 expression (Fig. 7E). The alterations in kcnh2 expression were not seen at earlier stages, becoming apparent only after the end of organogenesis (Fig. 7A). Expression of ncx1 was significantly lower in high dose individuals at 3 dph (p = 0.0023) (Fig. 7D) and was associated with the underdeveloped silent ventricle phenotype (Fig. 7E). However, in the pooled samples, although detecting an effect of dose, potentially reflecting the first time point (3 dpf) (Fig. 7C), two-way ANOVA did not detect a significant time-dose interaction for ncx1 (Table S2). No significant effects of dose were observed for wnt11, cacna1c and serca2.

**Figure 8:**
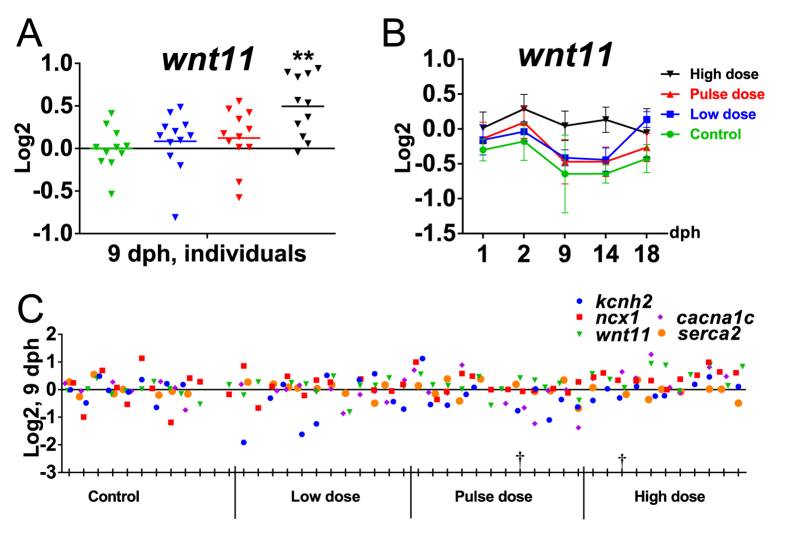
Expression of genes involved in EC coupling and *wnt11* in the larval exposure. (**A**) Expression of *wnt11* in individuals at 9 dph. (**B**) Expression of *wnt11* in pooled samples during larval exposure from 1 dph to 18 dph for all exposure groups and control during early larval phase. (**C**) Expression of *kcnh2, ncx1, cacna1c, serca2* and *wnt11* in individuals at 9 dph. Collectively, only *wnt11* varied significantly from control in individuals. ^†^Individual with A/V block. Statistical significant difference from control is indicated by *p < 0.05 and **p < 0.01.

In contrast to the embryonic exposure, there was no difference in kcnh2 expression in either pooled samples or individuals in the high dose group of the larval exposure, despite the observed AV block phenotype (Supplementary Fig. S12, Fig. 8). For wnt11, the two-way ANOVA showed an effect of dose, but no dose-time interaction (Table S2). Expression of wnt11 appeared to be dynamic during early larval development (Fig. 8A). While low and pulse dose follow the same dynamic expression for wnt11 as control, the high dose group appeared to have lost this regulation and instead showed a trend of overall increased expression at most time-points. This observation is consistent with significantly higher (p = 0.0006) levels of wnt11 in individuals at 9 dph (Fig. 8A). Further, the high dose group showed a trend of higher cacna1c levels at most time points (1–14 dph), and no differences in ncx1 and serca2 levels (Supplementary Fig. S12).

### Craniofacial defects are species-specific and not specific to oil source

The unusual craniofacial phenotypes observed here could either be a species-specific response of Atlantic haddock, or a novel activity associated with the composition of Heidrun crude oil. To make this distinction, we exposed zebrafish embryos to 50 mg/L dispersed Heidrun oil blend (compared to the haddock high dose of 0.6 mg/L), the same concentration tested in zebrafish embryos with other geologically distinct crude oils^28^,^29^,^48^. Although we did not measure PAH concentrations in this exposure, we used a standardized method to produce a high-energy water-accommodated fraction that was predicted to have ∑PAH in the range of 300–400 μg/L^21^,^22^. In contrast to what we observed in haddock, the zebrafish phenotypes were consistent with those observed with other crude oils. This level of Heidrun blend exposure produced high rates of pericardial edema (black arrow) and intracranial hemorrhage (white arrow) at 48 hours post fertilization (hpf) (Supplementary Fig. S13). Notably, these defects occurred at similar frequencies as for the same concentrations of crude oils from Alaska, Louisiana, and Iran^21^,^22^. Hearts in exposed embryos at 48 hpf showed poor looping (asterisk, Supplementary Fig. S13) and as for other crude oils, reduced contractility but not AV block arrhythmias (Supplementary Video S3). Importantly, exposed zebrafish at either 48 or 72 hpf did not show the same striking craniofacial phenotypes as haddock, but simply reduced lower jaw outgrowth as observed with other crude oils (Supplementary Fig. S13).

## Discussion

As expected, we observed more profound effects in haddock embryos when exposed to crude oil just after fertilization compared to exposure relatively late in embryogenesis^10^. Further, we show that despite a higher metabolic capacity, larval haddock are also susceptible toxicity from very low levels of dispersed crude oil. The most remarkable effect observed here is the dramatic craniofacial malformation following embryonic exposure. However, the severity and specificity of the craniofacial phenotypes are inconsistent with a simple secondary response to edema. In both the embryonic and larval exposures, we observed defects in cardiac function (heart rate and rhythm, contractility) that were detectable before or nearly coincidental with edema accumulation. Many aspects of these cardiac function phenotypes were also unexpected and specific to developmental stage. In particular, the effect oil exposure had on EC coupling gene expression was contradictory to the transcriptional responses to chronic pharmacological blockade previously shown for some ion channels^38^,^39^. A key question discussed in detail below is whether the craniofacial malformations represent a novel activity of crude oil, or can be explained by the known effects of crude oil-derived PAHs on intracellular calcium handling. Finally, these studies highlight the unique properties of the haddock eggshell that lead to oil droplet binding. Consequently, PAH uptake is prolonged and enhanced, with even very short exposure during early development causing severe defects.

### Cardiotoxic mechanisms in relation to cardiac phenotypes

In the embryonic exposure we observed two prominent cardiac phenotypes. In more severely impacted animals (e.g., HB), hearts were underdeveloped with silent ventricles. By contrast, animals with less severe gross phenotypes in the transient exposure and low dose groups had hearts with silent ventricles that appeared more normally developed. Depending on the exact circumstances, silent ventricle could reflect chemical blockade of ion channels, disruption of ion channel biosynthesis, or a defect in cardiac cell differentiation, either singly or in combination. In turn, the altered morphology could either be downstream of EC coupling defects or due to a more direct developmental defect. For example, the asystolic effect could be due in part to failed development of specialized conduction cells^49^. As an initial effort to make this distinction, we examined the expression of key EC coupling genes encoding likely direct targets of PAHs (Erg, Serca2) and other components that could be either contributory or compensatory (Ltcc, Ncx1). Our findings suggest at least a dual mechanism that involves ion channel blockade as well as biosynthesis.

An additional mechanism of cardiotoxicity is activation of AhR in heart muscle cells, as has been shown for some higher molecular weight PAHs (e.g., benzo[a]pyrene^50^,^51^,^52^, and the C4-alkylated phenanthrene, retene^16^). However, the role of cardiomyocyte AhR activation in crude oil toxicity is less certain. Studies in zebrafish have shown differential activation of AhR in cardiomyocytes depending on the state of weathering^20^,^22^, while myocardial Cyp1a induction was observed in Pacific herring (*Clupea pallasii*) exposed to the same source crude oil^14^. Nevertheless, AhR activation in cardiomyocytes is not required for crude oil cardiotoxicity^20^,^21^,^22^. Consistent with this, here we observed no myocardial induction of Cyp1a in either the embryonic or larval exposure. While we did observe endocardial Cyp1a induction in the larval exposure, consistent with prior studies, it was remarkable that in the most severely impacted embryos, there was no evidence of AhR activation anywhere in the heart. Therefore it is most likely that the effects on heart morphology are directly related to AhR-independent effects on EC coupling pathways from PAHs that are poor AhR ligands.

The reduced contractility of both chambers in the embryonic exposure is consistent with defects in intracellular calcium cycling^24^,^36^,^43^. Disruption of intracellular calcium levels also reduces cardiomyocyte proliferation, leading to smaller hearts^36^,^43^. Alternatively, reduced ventricular size could also result from effects on either cardiac neural crest cells or the second heart field. Indeed there is some overlap of cardiac phenotypes between oil exposure and ablation of either the second heart field or neural crest cells, including bradycardia and small ventricle^53^. While we do not have direct cellular evidence for effects on either the second heart field or cardiac neural crest cells, the remarkable craniofacial phenotypes potentially implicates direct effects of crude oil on neural crest cells in haddock embryos.

The persistence of the silent ventricle phenotype in the low dose group well past the end of exposure, and the uncoupling of the phenotype from *cyp1a* induction provide interesting insight into the complexity of PAH toxicokinetics. Remarkably, after 6 days in clean water (3 dph) in the low dose group, the silent ventricle phenotype actually occurred at a higher frequency. Given the return of *cyp1a* expression to control levels, one would assume that tissue PAHs were presumably eliminated by this point. However, at the end of exposure, *cyp1a* was not expressed in the cardiac tissues of haddock with an abnormal ventricular myocardium. While this may seem paradoxical (effects of PAHs in the absence of Cyp1a induction, the main indicator of the presence of strong AhR agonists in tissues), tricyclic PAHs that block cardiac ionic channels^24^ are poor inducers of Cyp1a. This suggests that specific cardiotoxic PAHs accumulating in ventricular myocardium are not as readily detoxified, as observed recently in olive flounder (*Paralichthys olivaceous*) embryos^54^, leading to effects that continue after Cyp1a expression decays. Alternatively, this delayed effect could represent mobilization of a pool of PAHs contained in the yolk mass as larvae from the low dose treatment absorbed yolk in the post-hatch stages, as described for other hydrophobic contaminants^55^. Along with prior studies demonstrating differential tissue distributions of individual PAHs in fish embryos^15^, these findings indicate that the toxicokinetics of PAH uptake and distribution are likely more complicated than previously appreciated. In conjunction with prior studies, our data suggest that individual PAHs within complex mixtures are differentially distributed in the embryo, with some PAHs being transported through the circulation to be metabolized in the liver, for example, with others accumulating in cardiac tissue and escaping liver detoxification. This likely reflects the uptake and circulatory pathways for PAHs in embryos, supported by the pattern of Cyp1a induction observed here. The bulk of uptake is likely to occur across the large surface area of the yolk sac, into the yolk sac sinus where circulation goes directly to the heart. This would give direct access of poor Cyp1a inducers to myocardial tissue and binding to molecular targets (i.e., ion channels) prior to delivery to the liver for metabolism. The stronger AhR ligands responsible for most of the Cyp1a inducing activity of crude oil do not have binding targets in the heart, and would thus bypass the heart to be delivered by the circulation to the liver, where they induce Cyp1a and are metabolized. This induction of Cyp1a in the liver by more potent AhR agonists hence would not serve to protect from cardiotoxic weak AhR agonists, because they have already bound their target in the heart.

Finally, most studies on ion channel gene transcription have focused on homeostasic regulation (i.e., in mature heart cells), and there is not always a clear link between mRNA and protein levels^37^. However, the few studies on transcriptional responses to pharmacological blockade demonstrated cognate up-regulation of genes for voltage-gated sodium and pacemaker channels^38^,^39^. In addition, studies of cardiac and skeletal muscle development generally show a strong correlation between increasing mRNA levels and functional ion channel proteins (e.g. refs 56 and 57). Thus, in the developing embryonic heart, it would be expected that the normal increase in mRNA level for *kcnh2*, for example, is tied to the continuous growth and addition of cardiomyocytes and assembly of new functional ion channels. Here we did not observe compensatory up-regulation in any EC coupling genes in exposed haddock, with an opposite response observed for *kcnh2* (potassium voltage-gated channel subfamily H member 2) and *ncx1* (sodium/calcium exchanger 1). Analysis of genetic mutations in *kcnh2* in zebrafish identified two mutant phenotypes, a collapsed silent ventricle associated with complete loss of function^32^, and a partial AV block with normal chamber morphology attributable to a weaker mutant allele^31^. Our ventricular phenotypes mirror these two classes of zebrafish mutant alleles closely. Reduced numbers or size of cardiomyocytes alone in the small ventricle phenotype is unlikely to explain the reduced levels of *kcnh2* and *ncx1*, because there was no effect on *cacna1c* and *serca2*. Moreover, despite the presence of chronic AV block in the larval exposure, there was no compensatory transcriptional response in EC coupling genes. This suggests that crude oil is acting through a dual mechanism by blocking both ion channel function^24^ and feedback to transcriptional regulation of ion channel genes. For example, regulation of sodium channel subunit genes was shown to be controlled by cytosolic Ca^2+^ levels^38^, thus, PAH related blockade of calcium channels^24^ could potentially disrupt a Ca^2+^ second messenger feedback mechanism controlling *kcnh2* compensatory transcription. Surprisingly, there are few papers describing the normal transcriptional regulation of *kcnh2*, and none to our knowledge assessing the effects of Erg blockade on *kcnh2* transcription.

The relationship between Ca^2+^ signaling and transcription of ion channel genes most likely reflects the recently characterized process of excitation-transcription (ET) coupling^58^. In ET coupling, the integration of extracellular signals with components of the EC coupling machinery is mediated through the phospholipase C/inositol 1,4,5-triphosphate (IP3) pathway, with IP3 receptors acting as an additional channel for release of Ca^2+^ from the sarcoplasmic reticulum and nuclear envelope^58^. If cells utilize a contiguous pool of Ca^2+^ within the sarcoplasmic reticulum and nuclear envelope, it is likely that depletion of this pool by crude oil-derived PAHs would impact both contractility and transcription.

### Potential relationship between cardiac and craniofacial defects

The links between intracellular calcium pools that regulate contractility and signaling to the nucleus provide a likely mechanism for the specific craniofacial defects we observed. First, the severe phenotypes in embryonically exposed animals cannot be explained solely by impaired circulation. The craniofacial defects resemble zebrafish mutants affecting several different developmental pathways that depend on intracellular calcium and could thus be impaired by crude oil. This includes midline signaling pathways, such as Wnt11, Hedgehog and Nodal pathways^59^,^60^. Although there were no major changes in *wnt11* mRNA levels in exposed embryos, the selective reduction of the basal neurocranium/palate (ethmoid plate) observed in the larval exposure may be connected to up-regulation of *wnt11*. However, palatal development involves signaling centers that utilize members of the fibroblast growth factor (FGF) family, Wnt family members in addition to Wnt11, as well as the planar cell polarity (PCP) pathway^61^,^62^,^63^. There are multiple avenues through which intracellular calcium impinges on these pathways, for example through phospholipase C (e.g., FGF receptors^64^), and both canonical Wnt and Wnt/Ca^2+^ signaling^65^,^66^,^67^. At the same time, there is a potential role for a Serca2 family member in midline signaling^68^. However, the expression of *serca* genes has not been described in detail in fish embryos, and *serca* genes were not expressed in neural crest cells in frog embryos^69^. In this light, it is intriguing that our craniofacial defects are also strikingly similar to those that arise from the lack of craniofacial muscle development or function^70^,^71^. Thus, abnormal formation of craniofacial cartilages could be secondary to effects of crude oil on the contractile apparatus of associated craniofacial muscles. Our findings in zebrafish embryos with total nominal oil load (and thus PAH concentrations) several orders of magnitude higher indicate that these craniofacial defects are specific to Gadids, and not due to a unique activity present in the Heidrun crude oil. Why these pathways are disrupted specifically in haddock remains to be determined, but most likely reflects differences in toxicokinetics, differential sensitivities of target proteins, or both.

### Implications of oil droplet binding for exposure duration and timing

The results here confirm and extend our previous findings^10^ and highlight the unusual negative consequences of oil droplet binding to the haddock eggshell. Accordingly, early embryonic exposure of haddock leads to more severe toxicity than might be expected from previous studies on other species and the nominal exposure concentrations used here (0.7–7 μg/L ∑PAH). This is emphasized by (1) the relatively severe effects of the pulse treatment, (2) defects arising from as little as 24 hours of transient early embryonic exposure, and (3) the differences between the embryonic and larval exposures, especially in regard to *cyp1a* induction.

The heterogeneity of phenotypes at a given oil concentration in the embryonic exposure probably reflects variability in internal dose due to variable accumulation of droplets, while the very uniform response in the larval exposure reflects primarily toxicity from dissolved PAHs. Both embryos and larvae were exposed to the same concentrations of oil droplets and dissolved PAHs, but peak *cyp1a* induction was 10-fold higher in embryos. Given that the single measurements for tissue PAH concentrations in embryos and larvae were close (only about 2.5-times higher in embryos) and taken at the end of exposure, the much higher early peak of *cyp1a* expression suggests that embryos probably accumulated higher peak tissue concentrations of PAHs at those earlier time points. Cyp1a metabolism could be more effective in larvae, but comparison to other species also indicates that haddock embryos receive a higher effective dose due to droplet binding. For example, Pacific herring embryos exposed to dissolved PAHs accumulated even higher tissue levels and showed cardiac defects (bradycardia and arrhythmia), but not the severe craniofacial phenotypes or gross malformations evident in the HB phenotype observed here^14^. Likewise, mahi mahi (*Coryphaena hippurus*) embryos exposed to physically dispersed oil containing total PAH of about 15 μg/L (oil droplets and dissolved combined) showed only cardiac defects and a lower level of *cyp1a* induction (~40-fold), and did not appear to bind oil droplets^27^. These interspecies comparisons therefore provide additional evidence for specific targets of crude oil toxicity in haddock craniofacial development.

Our findings also reveal how oil droplet binding influences the toxic impacts relating to exposure duration and developmental stage. The phenotypes in the pulse group are consistent with higher peak tissue concentrations at key sensitive time points relative to the low group. Although this is not reflected in the temporal pattern of *cyp1a* induction or final tissue PAH concentrations, which were indistinguishable between pulse and low groups, it is likely that *cyp1a* induction reflects an average or integrated response. Therefore, shorter exposure to higher levels of dispersed oil in early developmental stages can have a greater impact than expected based on an average PAH concentration. More importantly, oil droplet binding leads to prolonged effective exposure from even very brief interactions with dispersed oil, indicated by the relatively severe effects of the 24- and 48-hr exposures. These findings are especially important for risk assessment modeling, indicating that effects on haddock cannot be predicted simply on measured water concentrations of either dissolved or total PAHs in oil droplets.

## Conclusions

Our findings underscore the unique susceptibility of haddock embryos to dispersed crude oil toxicity as a likely consequence of oil droplet binding. Compounding this phenomenon, haddock show distinctive craniofacial malformations that could be linked to novel mechanisms of crude oil toxicity. In other species extracardiac morphological defects are secondary to cardiotoxicity, either through AhR agonism or disruption of EC coupling (or both). The atypical nature of haddock craniofacial defects and the absence of AhR agonism in cardiomyocytes strongly suggest that crude oil is having a direct effect on craniofacial development (e.g., midline signalling) in haddock. This unusual teratogenic effect of crude oil potentially highlights an emerging role for calcium signaling in midline and craniofacial development. Moreover, the negative effect of oil exposure on the expression of EC coupling genes is a newly identified mechanism of crude oil cardiotoxicity, most likely also involving the direct effects of tricyclic PAHs on ET coupling. Calcium signaling in development is complicated and challenging to dissect due to many interconnected roles in both excitable and non-excitable cells^72^,^73^. While oil-induced defects in heart development are simple to link to disruption of EC coupling, craniofacial defects could arise from disruption of calcium signaling in either excitable (e.g., skeletal muscle) or non-excitable cells (e.g., Wnt signaling in chondrocyte precursors). Moreover, ET coupling is only beginning to be characterized in detail^58^. Crude oil and specific compounds underlying the phenotypes observed here may be valuable tools for a greater understanding of the many roles intracellular calcium plays in vertebrate development and cellular function.

## Materials and Methods

### Animal collection, maintenance and exposure set up

A wild haddock broodstock population of 61 maturing individuals was collected February-March 2013 at spawning grounds in the Austevoll area, on the west coast of Norway, and kept in two outdoor 7000 L tanks at the Institute of Marine Research (IMR), Austevoll Research station. Haddock spawn voluntarily in captivity, and fertilized eggs could therefore be collected from the tanks, transferred to indoor egg incubators, and maintained at 7.8 °C until transmission to exposure tanks. At 2 days post fertilization (dpf) ~6000 eggs (embryonic exposure) and 13 dpf ~6000 eggs (larval exposure) were transferred into each of 16 circular exposure tanks (50 L) of green PE plastic (4 replicates for each treatment). The flow through the tanks was 32 L/hr, the water temperature 7.8 °C, and light regime was 12D:12L. Light for four replicate tanks was provided by the broad spectrum 2 × 36W Osram Biolux 965 (Munich, Germany, www.osram.com) dimmable fluorescent light tubes with 30 min smooth transitions between light and dark. From four days post hatching (dph), natural zooplankton, mainly copepod nauplii of *Acartia longiremis*, was harvested from the marine pond system “Svartatjern”^74^ and introduced as feed to the larvae.

The tanks were further supplemented with marine microalgae concentrate (Instant Algae, Nanno 3600, Reed Mariculture Inc., CA, USA) until termination of the experiments^75^.

### Oil exposure regime

The crude oil used was a weathered blend crude oil from the Heidrun oil field of the Norwegian Sea. The blend oil comes from 4 different formations that contain different oil types, both light paraffinic oils (0.83 g/ml) and heavy biodegraded oil (0.93 g/ml) and is exported as a heavy crude oil (0.89 g/ml). The Heidrun blend oil is believed to be representative for the characteristics of oil that may be found in the Lofoten area. The oil is artificially weathered by distillation to account for the fast evaporation that normally occurs after an oil spill at sea. This procedure^76^ is a simple one-stage distillation to vapor temperatures of 250 °C leaving a residue that corresponds to 2–7 days on the sea surface at about 10 °C ambient temperature, in this case causing an evaporative loss of 24% of the lighter compounds from the fresh crude oil, and it changes the oil density from 0.89 g/mL to 0.92 g/mL (SINTEF, 2004)^77^.

The principle of the exposure system and procedures for oil droplet generation are described in detail elsewhere^78^. The oil was pumped into the dispersion system using a HPLC pump (Shimadzu, LC-20AD Liquid Chromatograph Pump) with a flow of 5 μl/min together with a flow of seawater of 180 mL/min. The system generates an oil dispersion with oil droplets in the low μm range with a nominal oil load of 26 mg/L (stock solution). The exposure dose to the tanks was regulated by a parallel pipeline system with one line of flowing clean seawater and the other line containing a flow of the stock solution. The 2 pipelines were connected by a 3-way magnetic valve allowing water to be collected from both lines. Different dilutions were made by controlling the relative sampling time from the oil stock solution and clean water, respectively. The experimental setup consisted of three treatments and a control, each with four replicates: 60 μg oil/L (low dose), 600 μg oil/L (high dose), 600 μg oil/L for 2.4 hours in a 24 hour period (pulse dose), and no oil (control). The concentration of oil (measured PAHs) in the pulse tanks decreased to approximately zero before the next pulse (Supplementary Fig. S14). The oil dispersion doses were given by opening the magnetic valve for 27 seconds every minute in the high treatment group and 3 seconds in the low treatment group. The pulse treatment group received 27 seconds of oil stock solution every minute for 2.4 hours in each 24 hour period. Oil dispersions were delivered to each of the replicate exposure tanks at a flow rate of 30 mL/min and mixed into the main water supply of 500 mL/min. To avoid formation of oil film on the water surface in the tanks, an air valve was mounted in each tank to continuously blow air onto the water surface.

In the continuous embryonic exposure, the exposure was stopped after 7 days of exposure (10 dpf) and all surviving embryos were transferred to 16 new tanks with clean seawater for further monitoring. The embryos started to hatch at 11–12 dpf and 50% hatch was observed at 13 dpf (=0 dph). Hatching success for the various groups were 82% ± 2 in control, 78% ± 4 in low dose, 70% ± 9 in pulse dose and 50* % ± 18 in high dose (*significant different from control p ≤ 0.05). In the transient embryonic exposure, an aliquot of 300 eggs from all tanks were transferred to four tanks (one for each treatment and control) with clean seawater after 24 and 48 hours of initial exposure (3 and 4 dpf, Supplementary Fig. S14). The transient embryonic exposure was terminated at 2 dph. For the larval exposure, the exposure started at 0 dph and was stopped after 18 days (18 dph).

### Imaging of live embryos/larvae and measurements of cardiac function

Digital still micrographs of live larvae were obtained with an Olympus SZX-10 stereo microscope equipped with a 5 Mp resolution camera (Infinity 2–5c, from Lumenera) while video recordings where obtained using the same microscope and a Nikon SMZ-800, both with 1.2 Mp resolution video cameras (Unibrain Fire-I 785c). Image magnification was calibrated with a stage micrometer. BTV Pro 5.4.1 (www.bensoftware.com) was used to control the video camera. Video microscopy was performed at 1 and 3 dph (Continuous and Pulse embryo exposures, 60 larvae per treatment), 2 dph (Transient embryo exposure, 30 larvae per treatment) and 3 and 9 dph (Larval exposure, 48 larvae per treatment). Animals were immobilized in a glass petri dish filled with 3% methylcellulose and kept at 8 °C using a temperature controlled microscope stage. Length of ethmoid plate, mandible, area of edema and length, area and circularity of ventricle were measured using ImageJ (ImageJ 1.48r, National Institutes of Health, Bethesda, Maryland, USA, http://rsb.info.nih.gov/ij) with the ObjectJ plugin (https://sils.fnwi.uva.nl/bcb/objectj/index.html) from images as described in Fig. 5. At 0 dph for the embryonic exposure, the area occupied by edema fluid was quantified as the difference between the total area of the yolk sac and the area of the yolk mass, expressed as a percentage of total yolk sac area (Fig. 5). For larval exposure, we quantified edema accumulation at 9 and 18 dph with a similar approach, as the difference between the area occupied by the internal organs and the total pericardial and abdominal area. The assessment of edema at 9 dpf was based on presence/absence, not estimated edema area. ImageJ was also used to measure the ventricular and atrial diastolic (D) and systolic diameter (S) to estimate the fractional shortening (FS = (D − S)/D) (Fig. 5A,B). Atrial beats per minute (BPM) were also counted. Measurements from both images and videos were performed blind.

To quantify oil microdroplets on the eggshell we recorded PAH associated blue fluorescence using a Nikon AZ100 microscope equipped with a DAPI filter set (peak excitation 330–380 nm; emission >425 nm) and a Nikon Intensilight C-HGFI fluorescence light source. Pictures were taken using a 2x objective and 1x zoom and a QICAM monochrome camera (1392 × 1040 pixels, http://www.qimaging.com) resulting in images with a resolution of 0.237 pixels/μm. The microscope UV-light, camera exposure time and gain factor were initially adjusted to the high exposure group eggs so that oversaturation did not occure in the picture. These factors were then held constant (2 sec exposure time, gain 5) for all images. Each picture usually contained 6 eggs. Emission on the eggshell was estimated using Image J; each egg was encircled using the circular ROI (region of interest) tool and mean light intensity measured for each. If the eggshell had artifacts (e.g., small particles) that interfered with the measurement, these were excluded from the ROI before measurement.

### Analytical chemistry

Water samples (1 L) were taken from each exposure tank at the beginning of each experiment, preserved by acidification (HCl, pH < 2) and stored at 4 °C in the dark until further handling. The samples were extracted twice by partitioning to dichloromethane (30 mL) in a separatory funnel (2 min). Deuterated internal standards (naphthalene-d8, biphenyl-d8, acenaphtylene-d8, anthracene-d10, pyrene-d10, perylene-d12 and indeno(1,2,3-c,d)perylene-d12) were added prior to extraction to account for analyte loss during extraction. The combined extracts were concentrated by solvent evaporation prior to analysis by GC-MS. The following PAH compounds were measured: C0, C1-, C2-, C3-naphthalenes (N), anthracene (ANT), acenaphthylene (AC), acenaphtene (AE), fluorene (F), phenanthrene (P), C1-, C2-, C3-phenanthrene/anthracenes (P/A), C0, C1-, C2-, C3-dibenzothiophenes (D), fluoranthene (FL), pyrene (PY), benzo[a]anthracene (BAA), chrysene (C), benzo[bjk]fluoranthene (BKF), benzo[e]pyrene (BEP), benzo[a]pyrene (BAP), perylene (PER), benzo[ghi]perylene (BP), indeno[1,2,3-cd]pyrene (IND) and dibenzo[a,h]anthracene (DBA). The method is described in detail in^10^.

Pooled tissue samples of eggs (100 mg wet weight, corresponding to approximately 100 individuals) and larvae (100 individuals, approximately 200–250 mg wet weight) were collected at the final day of exposure. Duplicate samples were taken from each exposure tank, including controls. The samples were preserved by flash-freezing in liquid nitrogen and stored at −80 °C. Tissue samples were extracted by solid liquid extraction and purified by solid phase extraction prior to analysis by GC-MS/MS. The method is described in detail in Sørensen *et al.*^79^. Briefly, after addition of internal standards (100 ng/g naphthalene-d8, biphenyl-d8, acenaphtylene-d8, anthracene-d10, pyrene-d10, perylene-d12 and indeno(1,2,3-c,d)perylene-d12) the samples were homogenized in 2 mL dichloromethane-hexane (1:1, v/v), followed by addition of sodium sulphate (150 mg), vortex extraction (30 sec) and centrifugation (2000 rpm, 2 min). The supernatant was collected and the extraction repeated twice more. The combined organic extract was concentrated to ~1 mL prior to clean-up by SPE (Agilent Bond Elut SI, 500 mg). The SPE columns were conditioned with hexane and the extract eluted with 6 mL 10% dichloromethane in hexane. Immediately prior the analysis, the purified extract was concentrated to 100 μL under a gentle stream of N_2_.

### RNA collection and preparation

Embryos (before hatching) and larvae (after hatching) were collected ten times from 3 dpf to 3 dph, in addition to an initial sample at 2 dpf. In the larval exposure seven samples were collected from 0 dph to 22 dph, in addition to an initial sample at 0 dph. For the transient embryo exposure, RNA samples were collected at the end of the experiment (2 dph). All animals collected for RNA extraction were imaged under a microscope before they were frozen in liquid nitrogen and stored at −80 °C. Two pools of embryos and larvae from each tank were collected at all sampling times for total RNA extraction. In addition individual larvae were sampled and preserved in RNA*later* Stabilization Solution (Life Technologies Corporation) at 3 dph (embryonic exposure) and 9 dph (larval exposure).

Total RNA was isolated from frozen pools with varying amount of embryos (15–25) and pools of ten larvae using Trizol reagent (Invitrogen, Carlsbad, California, USA), according to procedures provided by the manufacturer which included a DNase treatment step using a TURBO DNA-*free* kit (Life Technologies Corporation). Total RNA from single larvae from 3 dph (embryonic exposure) and 10 dph (larval exposure) were extracted using RNeasy micro kit (QIAGEN Sample and Assay Technologies) according to procedures provided by the manufacturer. For both embryonic and larval exposure 12 larvae equally representative of each replicate tank analyzed were individually extracted. For the embryonic exposure, additional larvae with the hunchback or Darth Vader phenotype were extracted to verify if significant changes in gene expression of *ncx1* and *kcnh2* observed in the initial sample of 12 individuals were robust. All samples were homogenized in their respective lysis buffer 2 × 20 seconds at 5000 rpm using a Precellys 24 apparatus (Precellys). The amount of RNA was quantified using a Nanodrop spectrophotometer (NanoDrop Technologies, Wilmington, DE, USA), and quality checked using a 2100 Bioanalyzer (Agilent Technologies, Santa Clara, CA). cDNA was subsequently generated using SuperScript VILO cDNA Synthesis Kit (Life Technologies Corporation), according to the manufacturer’s instructions. The cDNA was normalized to obtain a concentration of 50 ng/μL.

### Real time qPCR

Specific primers and probes for real-time, qPCR analysis of Atlantic haddock, *cyp1a* and the technical reference *ef1a* and *kcnh2*, *ncx1, serca2, cacna1c and wnt11* mRNAs were designed with Primer Express software (Applied Biosystems, Carlsbad, California, USA) and Eurofins qPCR probe and primer design software (Eurofins Scientific, Ebersberg, Germany), respectively, according to the manufacturer’s guidelines. Primer and probe sequences are given in Supplementary Table S3. TaqMan PCR assays were performed in duplicate, using 384-well optical plates on an ABI Prism Fast 7900HT Sequence Detection System (Applied Biosystems, Carlsbad, CA, USA) with settings as follows: 50 °C for 2 min, 95 °C for 20 s, followed by a 40 cycles of 95 °C for 1 s and 60 °C for 20 s. Duplicates with standard deviation^2^ (SD^2^) ≤ 0.05 were either rerun or eliminated from the dataset. No template, no reverse transcriptase enzyme control and genomic DNA controls were included. For each 10 μl PCR reaction, a 2 μl cDNA 1:40 dilution (2.5 ng) was mixed with 200 nM fluorogenic probe, 900 nM sense primer, 900 nM antisense primer in 1xTaqMan Fast Advanced Master Mix (Applied Biosystems, Carlsbad, California, USA). Gene expression data for *kcnh2, ncx1, wnt11, cacna1c* and *serca2* in pools were calculated relative to the start sample (2 dpf and 1 dph for embryonic and larval exposure, respectively) using the ΔΔΔCt method generating reference residuals from *ef1a* (technical reference) and *serca2* (biological reference) in the embryonic and larval exposure, as described in detail in Edmunds *et al.*^80^. While gene expression data from individuals at 3 dph (embryonic exposure) and *cyp1a* in pools was calculated relative to control or start point, respectively, using the ΔΔCt method as described in detail in Bogerd *et al.*^81^.

### Cyp1a immunofluorescence

Haddock larvae were collected on the day of hatch initiation, one day after termination of exposure, fixed overnight in 4% paraformaldehyde buffered in PBS, then rinsed in PBS and transferred to methanol for storage. Whole mount labeling with anti-Cyp1a monoclonal C10-7 (cod antigen) and AlexaFluor 488-labeled anti-mouse IgG_3_ was carried out as described elsewhere and imaged on a Zeiss LSM-Pascal confocal system^13^. Twelve larvae from each treatment group were labeled and examined, with representative images collected from three randomly selected individuals.

### Histology

Six larvae from each replicate were pooled and fixed in 4% PBS buffered paraformaldehyde for 24 hrs at 4.0 °C, processed using Histokinette 2000 (Reichert-Jung), and embedded in paraffin wax within three days to preserve the RNA and tissue morphology. Sample preparation was always performed under RNase free conditions. Serial sectioning (3 μm) of larvae was performed for morphological analysis or *in situ* hybridization using a Leica RM 225 microtome (Leica Microsystems).

For *in situ* hybridization (ISH), the Atlantic haddock *cyp1a* cDNA was amplified using PCR containing Sp6 and T7 primers in the forward and reverse primers, respectively (Cyp1a_FW_Sp6: 5′-ATTTAGGTGACACTATAGCATCTTCCAGATCCAGATCG-3′ Cyp1a_RV_T7: 5′-TAATACGACTCACTATAGGGCATGAACCTCTTCATGGTGG-3′). The PCR product of 501 bp was used as a template for synthesizing the sense and antisense cRNA probes by the Sp6 and T7 RNA polymerase, respectively. The digoxigenin (DIG) labeling was performed using the DIG-AP RNA labeling kit (Roche Molecular Biochemicals) following the manufacturers protocol.

The *in situ* hybridization was carried out as described by Weltzien *et al.*^82^ with some modifications^83^. Hybridization was always carried out with sense and antisense probes on adjacent sections, and under RNase free conditions.

### Alcian blue staining

Three days post hatching, larvae from the embryonic exposure were fixed overnight in 4% PBS-buffered paraformaldehyde, rinsed in PBS and transferred to methanol for storage at −20 °C. After re-hydration, 20 larvae per treatment were stained with Alcian blue, dissected and imaged as described elsewhere^21^.

### Zebrafish exposures

Zebrafish embryos were collected from AB adult fish maintained as described elsewhere^21^. A high-energy water accommodated oil fraction (HEWAF) of weathered Heidrun oil was prepared in zebrafish system water at 50 mg/L using a stainless steel commercial blender^21^. Embryos were exposed to whole HEWAF (containing oil droplets) in 100-mm glass petri dishes at a density of 2 embryos/mL beginning at ~50% epiboly to 72 hpf without renewal (three replicates of 80 embryos each for control and 100% HEWAF). Embryos were scored for gross abnormalities, pericardial edema and intracranial haemorrhage^21^ at ~48 hpf and craniofacial phenotypes at 72 hpf, with random embryos selected for imaging at 48 and 72 hpf using a Nikon SMZ-800 stereomicroscope with a Unibrain Firewire-800 camera (Fire-i 780c) and BTV Pro 5.4.1 software.

### Statistics

Statistical analysis was performed with GraphPad Prism, version 6 (GraphPad Software Inc., 1996, La Jolla, California, USA). Significant differences in structural and functional measurements, phenotypic characterization, PAH related fluorescence intensity on the eggshell and gene expression in individual samples were tested with one-way ANOVA using the Tukey-Kramer multiple comparison. Gene expression in pooled samples was analyzed using Two-way ANOVA, with Dunnet’s multiple comparison used in post-hoc tests in cases where there was a significant time-dose interaction. The level of significance was set at p < 0.05 unless otherwise stated.

### Ethics Statement

All animal experiments within the study were approved by NARA, the governmental Norwegian Animal Research Authority (http://www.fdu.no/fdu/, reference number 2012/275334-2). All methods were performed in accordance with approved guidelines. All embryos sampled were frozen in liquid nitrogen. All larvae were euthanized using 500 mg/L MS-222 (Tricaine methanesulfonate, TS 222, Sigma-Aldrich) when sampling and at termination of the experiment to achieve immediate death. The animals were monitored daily, and any dead larvae were removed. The Austevoll Aquaculture Research station has the following permission for catch and maintenance of Atlantic haddock: H-AV 77, H-AV 78 and H-AV 79. These are permits given by the Norwegian Directorate of Fisheries. Furthermore, the Austevoll Aquaculture Research station has a permit to run as a Research Animal facility using fish (all developmental stages), with code 93 from the national IACUC; NARA.

## Additional Information

**How to cite this article**: Sørhus, E. *et al.* Crude oil exposures reveal roles for intracellular calcium cycling in haddock craniofacial and cardiac development. *Sci. Rep.* **6**, 31058; doi: 10.1038/srep31058 (2016).

## Supplementary Material

Supplementary Movie S1

Supplementary Movie S2

Supplementary Movie S3

Supplementary Information

## Figures and Tables

**Table 1:** Craniofacial phenotypes after transient and continuous embryonic exposure.

Exposure	STAGE	Group	BD^1^, %	JB, %	DV, %	HB, %	BE % of control
Transient exposure	24 h, 2 dph (13 dpe^2^)	Control	0	0	0	0	—
		Low dose	0	0	0	0	—
		Pulse dose	0	0	0	0	—
		High dose	7	0	3	0	—
	48 h, 2 dph (12 dpe)	Control	0	0	0	0	—
		Low dose	0	0	0	0	—
		Pulse dose	0	0	0	0	—
		High dose	17	0	10	3	—
Continuous embryonic exposure	E0 dph (3 dpe)	Control	—	—	—	—	100 ± 21^a^
		Low dose	—	—	—	—	74 ± 23^b^
		Pulse dose	—	—	—	—	86 ± 13^c^
		High dose	—	—	—	—	86 ± 16^c^
	3 dph (6 dpe)	Control	1 ± 3^a^	0^a^	0^a^	0^a^	—
		Low dose	17 ± 7^b^	15 ± 18^b^	2 ± 3^b^	0^a^	—
		Pulse dose	28 ± 8^c^	2 ± 3^a,c^	27 ± 19^c^	0^a^	—
		High dose	15 ± 9^b^	4 ± 5^c^	23 ± 10^c^	54 ± 19^b^	—

^1^BD, bulldog; JB, jaw breaker; DV, Darth Vader; HB, hunchback; BE, distance between eyes.

^a–d^Letters indicate significant differences between groups (p = <0.05) (groups with same letters are not significantly different from each other).

^2^Dpe = days post exposure stop.

**Table 2:** Craniofacial outgrowth in larval exposure.

Exposure	STAGE	Group	EP^3^, μm	M, μm	EP/M, %
Larval exposure	2 dph	Control	135 ± 17^a^	417 ± 30^a^	32 ± 4^a^
		Low dose	122 ± 17^b,c^	395 ± 66^b^	31 ± 4^a^
		Pulse dose	132 ± 20^a,b^	411 ± 24^a^	32 ± 5^a^
		High dose	118 ± 16^c^	388 ± 28^b^	30 ± 4^a^
	9 dph	Control	219 ± 21^a^	586 ± 45^a^	37 ± 5^a^
		Low dose	197 ± 27^b^	557 ± 44^b^	35 ± 5^a,b^
		Pulse dose	199 ± 29^b^	554 ± 47^b^	36 ± 5^a,b^
		High dose	170 ± 28^c^	495 ± 39^c^	34 ± 5^b^
	18 dph	Control	314 ± 49^a^	2442 ± 252^a^	38 ± 5^a^
		Low dose	295 ± 55^a,b^	2324 ± 262^a^	37 ± 6^a^
		Pulse dose	263 ± 63^b^	2216 ± 310^b^	34 ± 5^a^
		High dose	151 ± 39^c^	1812 ± 165 ^c^	25 ± 5^b^

^3^EP, ethmoid plate; M, mandible.

^a–d^Letters indicate significant differences between groups (p = <0.05) (groups with same letters are not significantly different from each other).

**Table 3:** Characterization of cardiac function and morphology.

Exposure	STAGE	Group	Edema area, %	BPM^4^	SV, %	VFS, % SV incl.	VFS, % SV excl.	AFS, %	VL % of control	VA % of control	VC % of control	% A/V block
Transient exposure	24 h, 2 dph (13 dpe)	Control	8 ± 5^a^	57 ± 6	0	18 ± 4^a^	18 ± 4^a^	—	—	—	—	0
		Low dose	9 ± 5^a^	57 ± 7	0	16 ± 5^a^	16 ± 5^a^	—	—	—	—	0
		Pulse dose	10 ± 7^a^	57 ± 5	0	16 ± 5^a^	16 ± 5^a^	—	—	—	—	0
		High dose	7 ± 4^a^	56 ± 5	20	9 ± 5^b^	12 ± 5^b^	—	—	—	—	0
	48 h, 2 dph (12 dpe)	Control	10 ± 5^a^	57 ± 8	0	17 ± 4^a^	17 ± 4^a^	—	—	—	—	0
		Low dose	11 ± 7^a^	59 ± 7	17	13 ± 8^a,b^	16 ± 6^a,b^	—	—	—	—	0
		Pulse dose	9 ± 4^a^	56 ± 5	7	14 ± 6^a^	16 ± 5^a,b^	—	—	—	—	0
		High dose	17 ± 12^b^	54 ± 5	23	9 ± 7^b^	13 ± 6^b^	—	—	—	—	0
Continuous embryonic exposure	9 dpf	Control	†10 ± 12^a^	26 ± 3^a^	2 ± 4^a^	—	—	—	—	—	—	—
		Low dose	†28 ± 18^b^	25 ± 5^a^	14 ± 5^b^	—	—	—	—	—	—	—
		Pulse dose	†43 ± 32^c^	26 ± 4^a^	44 ± 21^c^	—	—	—	—	—	—	—
		High dose	†70 ± 20^d^	20 ± 6^b^	49 ± 15^c^	—	—	—	—	—	—	—
	0 dph (3 dpe)	Control	7 ± 6^a^	41 ± 6^a^	5 ± 5^a^	17 ± 7^a^	18 ± 6^a^	25 ± 6^a^	100 ± 21^a^	100 ± 32^a^	100 ± 5^a^	0
		Low dose	16 ± 9^b^	42 ± 6^a^	13 ± 8^b^	15 ± 7^a,b^	16 ± 6^a^	25 ± 7^a^	89 ± 20^b^	84 ± 33^b^	99 ± 4^a^	0
		Pulse dose	31 ± 14^c^	41 ± 7^a^	30 ± 15^c^	12 ± 9^b^	15 ± 7^a^	24 ± 6^a^	64 ± 16^c^	49 ± 21^c^	93 ± 6^b^	0
		High dose	31 ± 12^c^	34 ± 11^b^	54 ± 28^d^	4 ± 6^c^	9 ± 6^b^*	16 ± 8^b^	51 ± 21^d^	35 ± 23^d^	85 ± 14^c^	0
	3 dph (6 dpe)	Control	—	59 ± 7^a^	2 ± 3^a^	17 ± 6^a^	17 ± 5^a^	21 ± 7^a^	—	—	—	0
		Low dose	—	59 ± 6^a^	57 ± 25^b^	7 ± 7^b,d^	12 ± 6^b^	17 ± 7^b^	—	—	—	0
		Pulse dose	—	58 ± 8^a^	29 ± 17^c^	12 ± 8^c^	15 ± 6^a,c^	17 ± 6^b^	—	—	—	0
		High dose	—	46 ± 10^b^	72 ± 20^d^	3 ± 6^d^	14 ± 4^b,c^*	11 ± 7^c^	—	—	—	0
Larval exposure	2 dph	Control	—	73 ± 10^a^	0	16 ± 4^a^	—	—	—	—	—	4 ± 8^a^
		Low dose	—	77 ± 7^a^	0	15 ± 5^a^	—	—	—	—	—	2 ± 4^a^
		Pulse dose	—	78 ± 10^a^	0	16 ± 5^a^	—	—	—	—	—	2 ± 4^a^
		High dose	—	77 ± 11^a^	0	16 ± 6^a^	—	—	—	—	—	2 ± 4^a^
	9 dph	Control	9 ± 3^a^	105 ± 12^a^	0	17 ± 3^a^	—	—	—	—	—	0 ± 0^a^
		Low dose	12 ± 4^b^	104 ±19^a^	0	17 ± 4^a^	—	—	—	—	—	2 ± 4^a^
		Pulse dose	12 ± 4^b^	110 ± 13^a^	0	17 ± 4^a^	—	—	—	—	—	8 ± 7^b^
		High dose	13 ± 4^b^	106 ± 15^a^	0	21 ± 4^b^	—	—	—	—	—	17 ± 12^c^
	18 dph	Control	7 ± 5^a^	—	—	—	—	—	—	—	—	—
		Low dose	15 ± 16^a^	—	—	—	—	—	—	—	—	—
		Pulse dose	13 ± 11^a^	—	—	—	—	—	—	—	—	—
		High dose	32 ± 13^b^	—	—	—	—	—	—	—	—	—

^4^BPM, beats per minute; SV, silent ventricle; VFS, ventriclular fractional shortening; AFS, atrial fractional shortening; VL, ventricular length; VA, ventricular area; VC, ventricular circularity; A/V atrioventricular. Dpe = days post exposure stop.

^a–d^Letters indicate significant differences between groups (p = <0.05) (groups with same letters are not significantly different from each other).

VFS measurements were not performed in animals with severely underdeveloped ventricles.

^†^Incidence of edema, not area.

*Few animals with beating ventricles.
