# Supplementary material for: Crude oil exposures reveal roles for intracellular calcium cycling in haddock craniofacial and cardiac development
Source: Sci Rep. 2016 Aug 10;6:31058. doi: 10.1038/srep31058 (PMC4979050; doi:10.1038/srep31058)
Supplement: Supplementary Information [file srep31058-s4.pdf]

Supplementary Information

Crude oil exposures reveal roles for intracellular calcium cycling in haddock craniofacial and cardiac development

Elin Sørhus, John P. Incardona, Ørjan Karlsen, Tiffany Linbo, Lisbet Sørensen, Trond Nordtug, Terje van der Meeren, Anders Thorsen, Maja Thorbjørnsen, Sissel Jentoft, Rolf B. Edvardsen and Sonnich Meier

Supplementary Figure S1: PAH profile. PAH profile at end of exposure in (A) water and (B) tissue in embryonic exposure, and (C) water and (D) tissue in larval exposure.

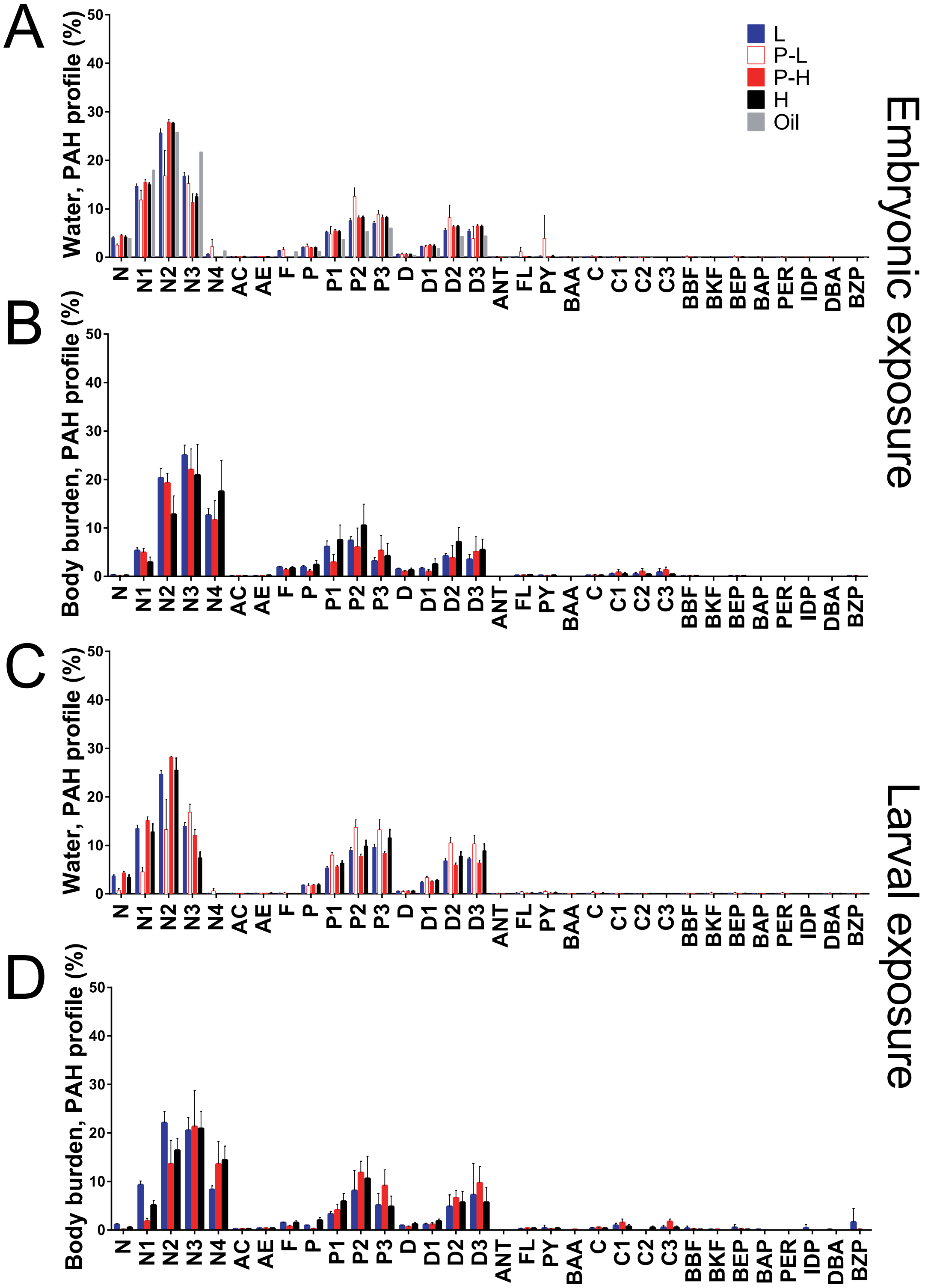

Supplementary Figure S2: Accumulation of oil droplets on eggshells. Control (left panels) vs oil-exposed embryos (right panels) after 24 (A) and 72 (B) hours of exposure. Areas with higher concentrations of dispersed oil droplets are indicated by arrows.

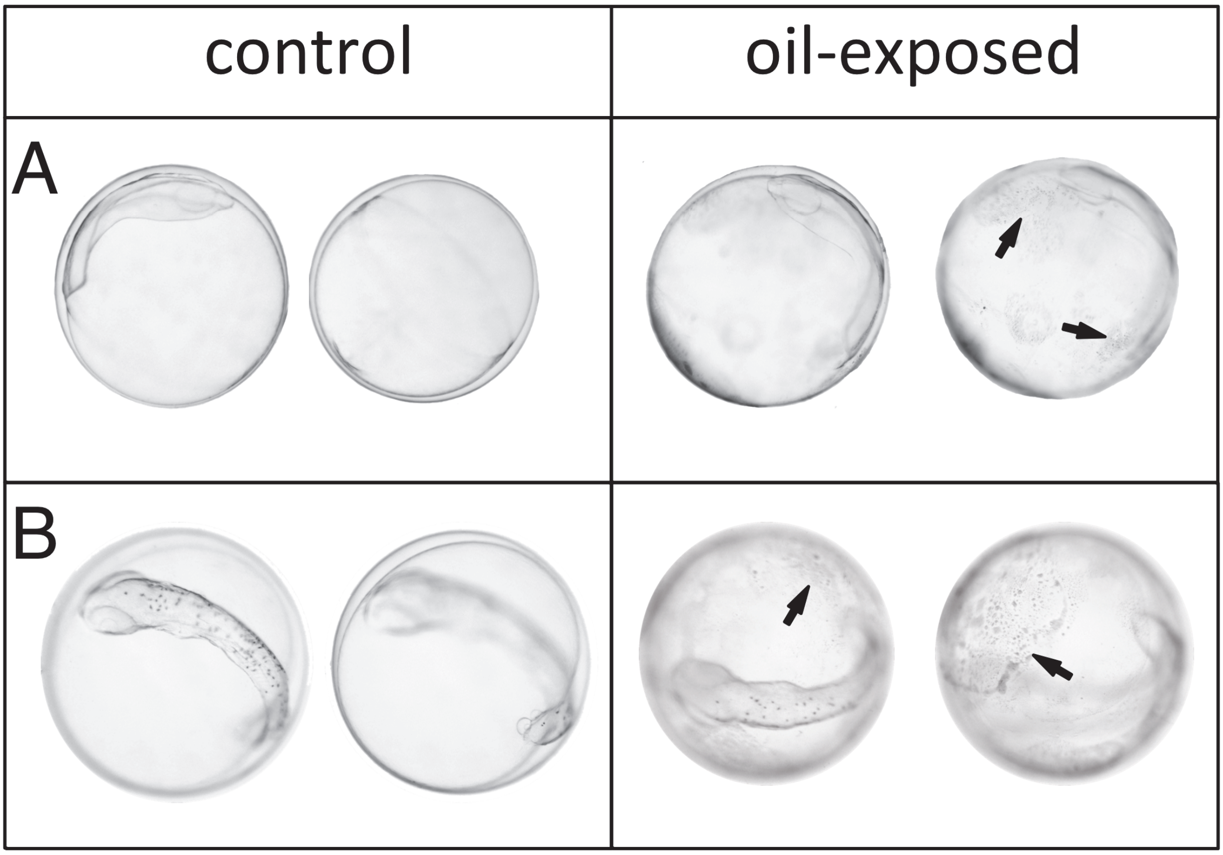

Supplementary Figure S3: Reduced toxicity and ∑PAH levels in outlier tanks. Log_2 normalized *cyp1a* mRNA levels in all low dose tanks (A) and pulse dose tanks (B) during and after exposure. (C) Dose relationship between ∑PAH and *cyp1a* mRNA levels X-axis shows log_{10} normalized ∑PAH data with corresponding log_2 normalized *cyp1a* mRNA levels on the y-axis. Individual tank identities are indicated for each point; P1 and L3 tanks cluster as a separate dose.

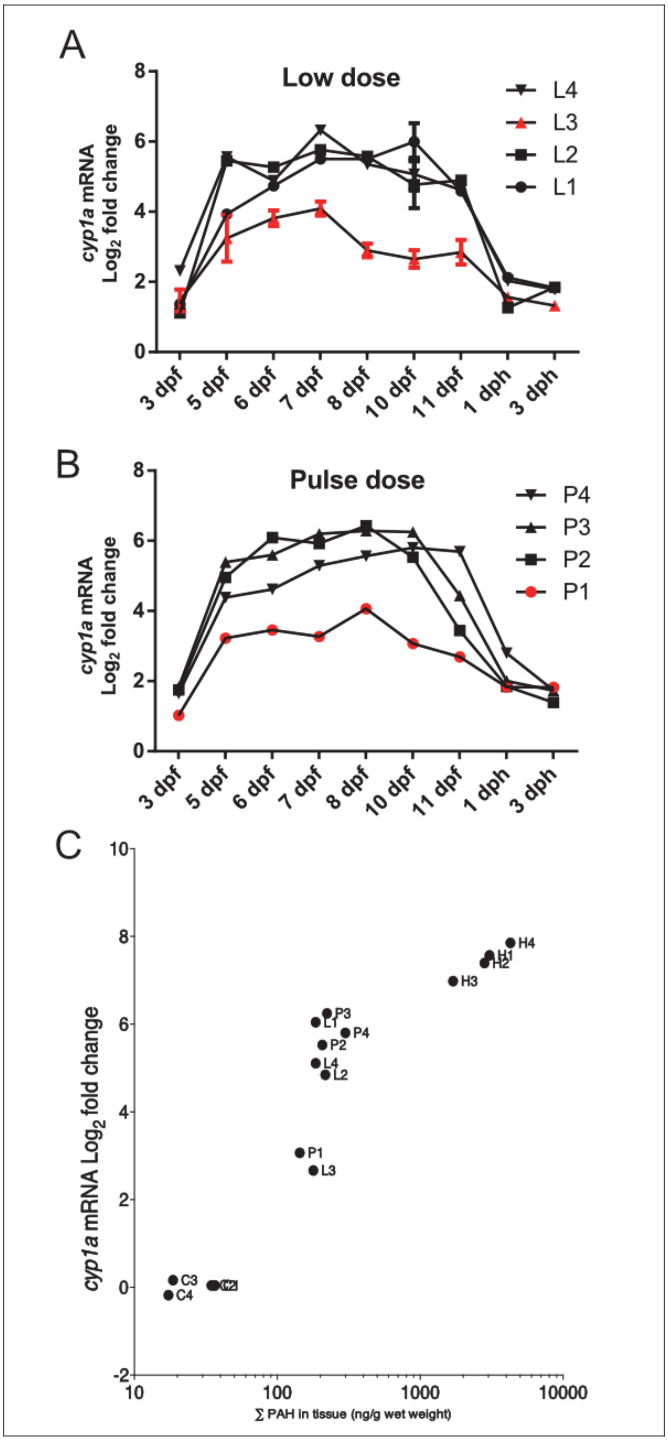

Supplementary Figure S4: Relative expression of *cyp1a* in embryonic (A) and larval (B) exposures. Expression is normalized to the expression at exposure start (2 dpf and 0 dph in embryo and larval exposure, respectively). Asterisks indicate statistical significance difference from control larvae, $p < 0.01$ = **.

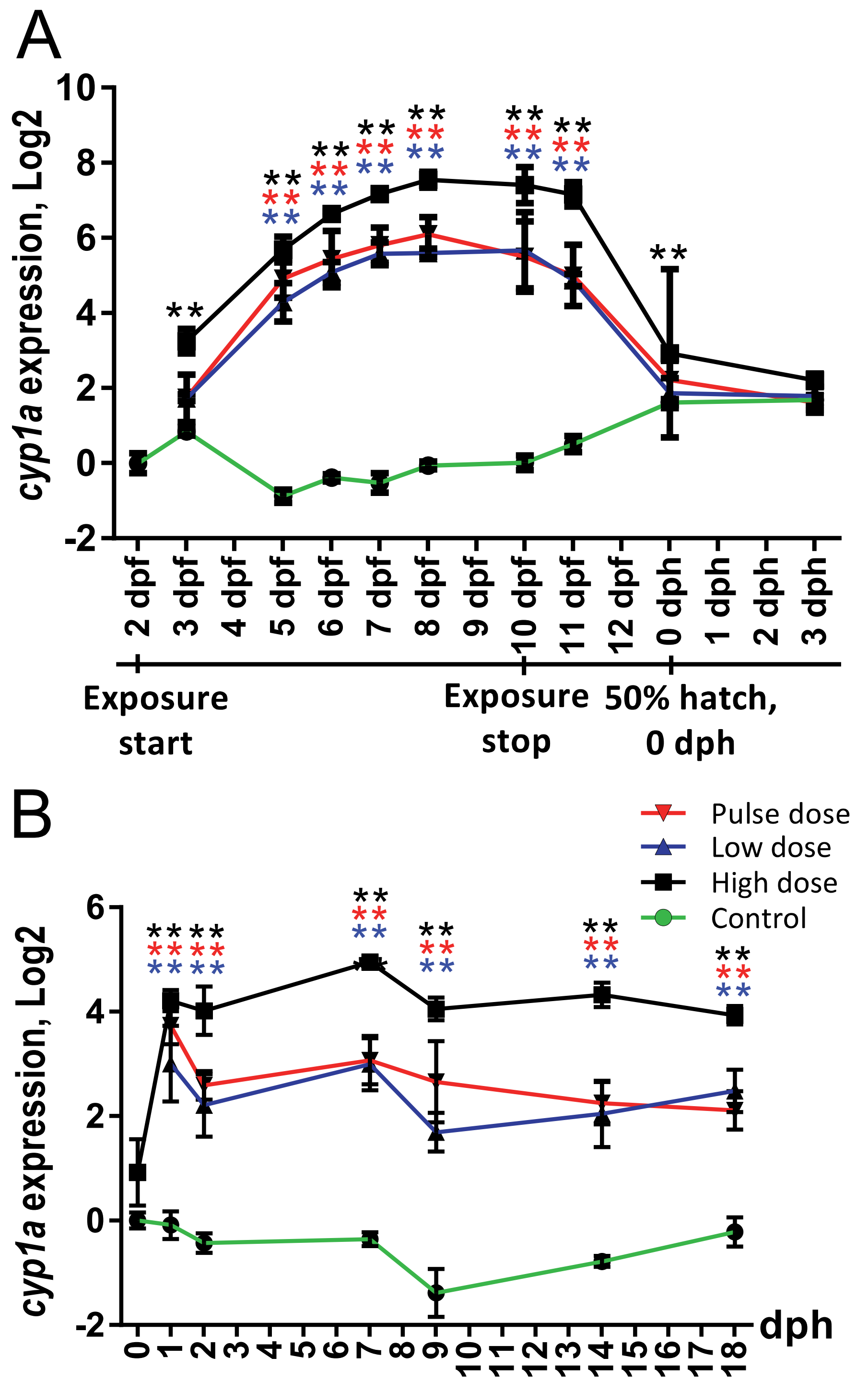

Supplementary Figure S5: Tissue distribution of Cyp1a in embryonic exposure. Whole mount immunofluorescence detection of Cyp1a protein in newly hatched larvae one day post exposure in (A) control, (B) low dose, (C) high dose. (D) Higher magnification of the heart in a high dose animal. (A-C) Filled arrows indicate the liver bud. (D) Open arrowheads and unfilled arrow in mark the myocardium and chamber lumen, respectively; arrow indicates epidermis. (A'-D') Corresponding light microscopic images. Scale bar: 200 µm. Anterior left, dorsal top.

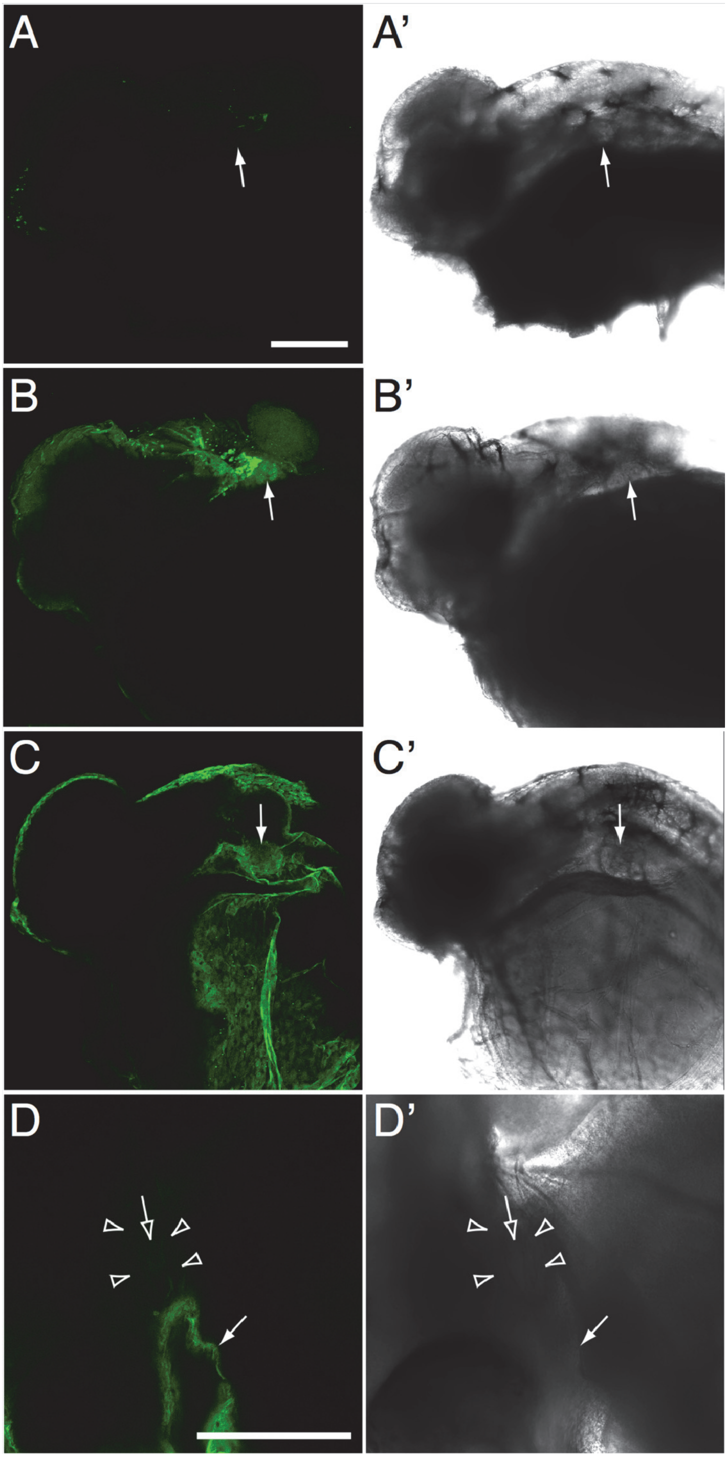

Supplementary Figure S*6*. *In situ* hybridisation of cyp1a in larval exposure. Location of *cyp1a* after one day of exposure (1 dph) in (A) control (saggital plane), low dose (saggital plane) and high dose larvae (frontal plane). (B) Location of *cyp1a* after 18 days of exposure (18 dph) in control (saggital plane), low dose (saggital plane) and high dose larvae (saggital plane). Expression of *cyp1a* was observed in cardiac endothelium (h), cardiac myocardium (m), epidermis (e), kidney, in the liver or the cells surrounding the blood vessels in the liver (l), intestine (i), gall bladder (b), brain (n) and gills (g) and sinusoid lining cells (s). Scale bars: 0.1 mm.

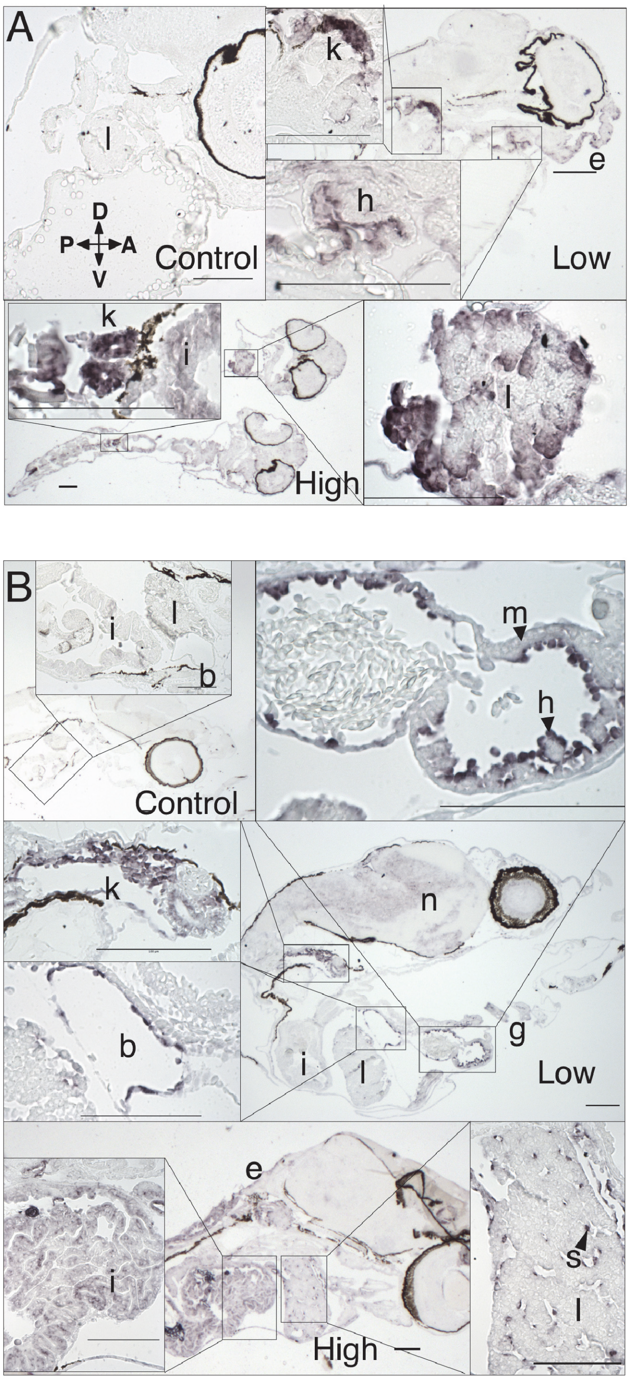

Supplementary Figure S7: Correlation between coloboma and hypotelorism. Several oil exposed individuals suffered from coloboma (filled arrow head) and the same individuals also showed a higher grade of hypotelorism (shortening of length between the eyes) (open arrow head).

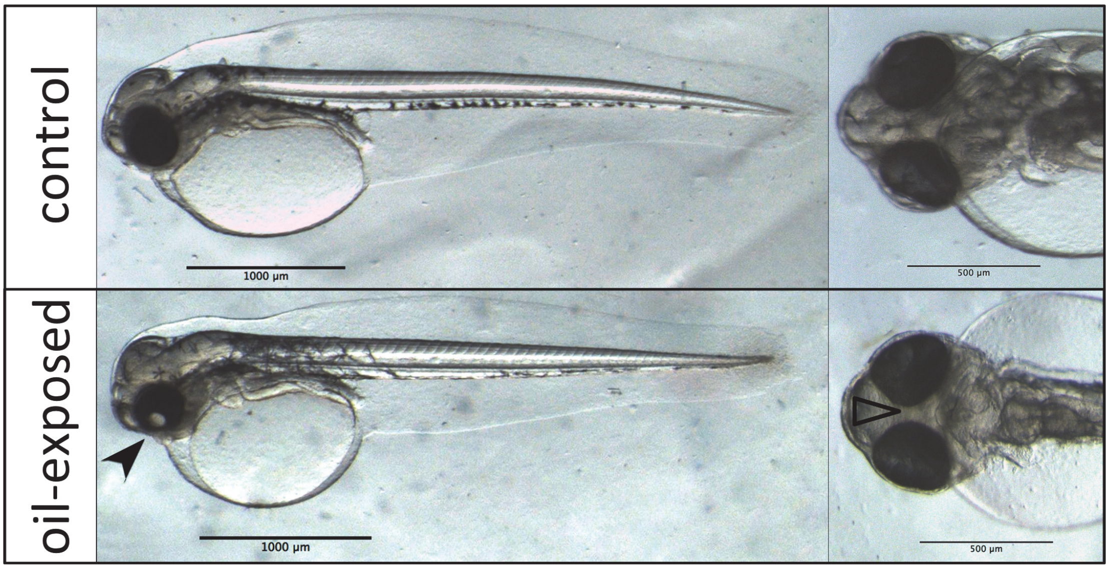

Supplementary Figure S8: Shortening of ethmoid plate. (A) Shortening of ethmoid plate (filled arrow head) and increased amount of edema (asterisk) after 9 days of exposure during early larval period in control, low dose, pulse dose and high dose. (B) Growth of the ethmoid plate in control and exposed animals from 1 dph to 21 dph. Significant difference (p = < 0.05) between groups is indicated by letters (i.e., groups with same letters are not significantly different from each other). Scale bar = 1 mm.

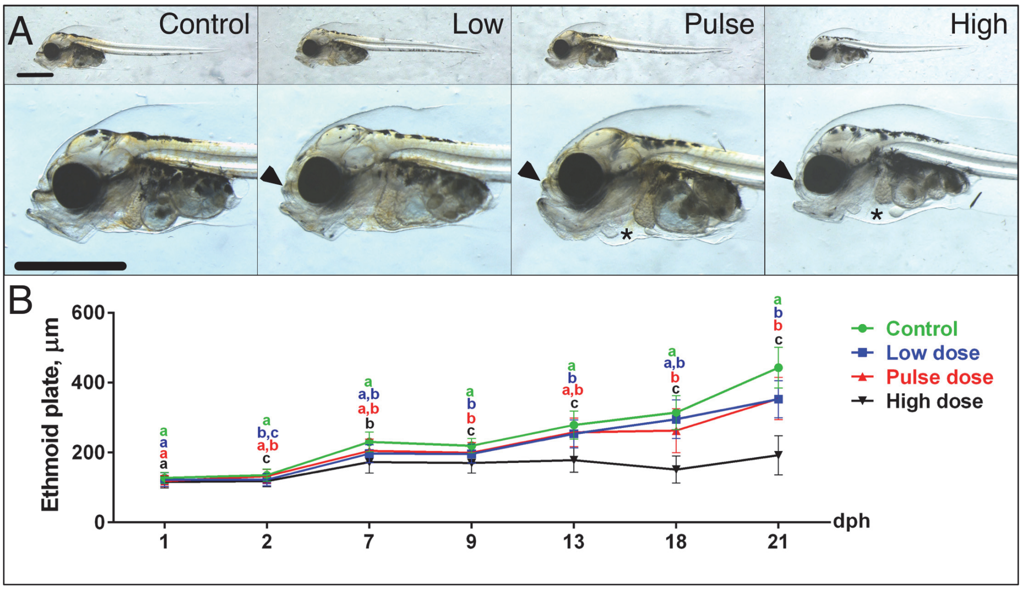

Supplementary Figure S9: Severity of edema in phenotypes. (A) The severity of edema in the four distinct phenotypes (BD = bulldog, JB = Jaw breaker, DV = Darth Vader and HB = hunchback) and in animals with no distinct phenotype. Severity of edema was graded from 0 (no edema) to 3 (severe edema) (B) Examples of phenotypes with various amount of edema.

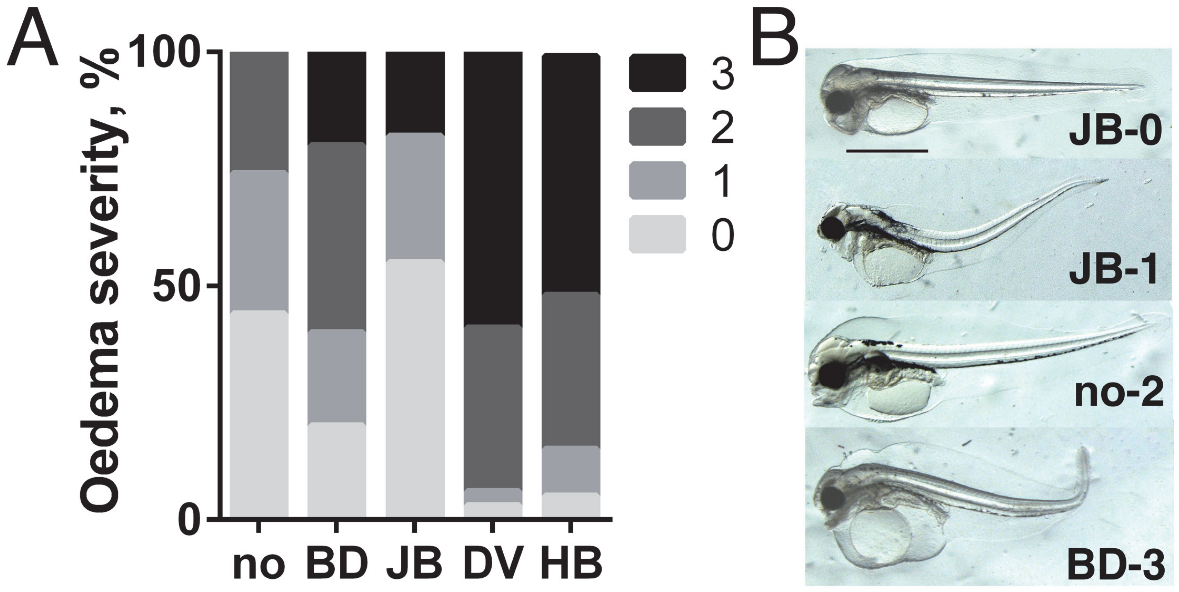

Supplementary Figure S10: Measurement of cardiac function. Measurements were performed on 2 dph larvae exposed for 24 and 48 hours in early embryo phase (pink rectangle), 0 dph and 3 dph larvae exposed throughout organogenesis and 2 and 9 dph exposed during larval phase. For means, standard deviation and significance for (A) see Table 1. (A) Fractional shortening (FS) of ventricle for embryonically exposed larvae with and without silent ventricles (SV) included, atrial FS for 0 dph and 3 dph (embryonic exposure) and ventricular FS for animals exposed in larval stages, no silent ventricles were observed here. (B) Ratio of atrial beats per minute vs ventricular beats per minute for 9 dph larvae exposed during larval stages, revealing individuals bypassing ventricular beats in red rectangle.

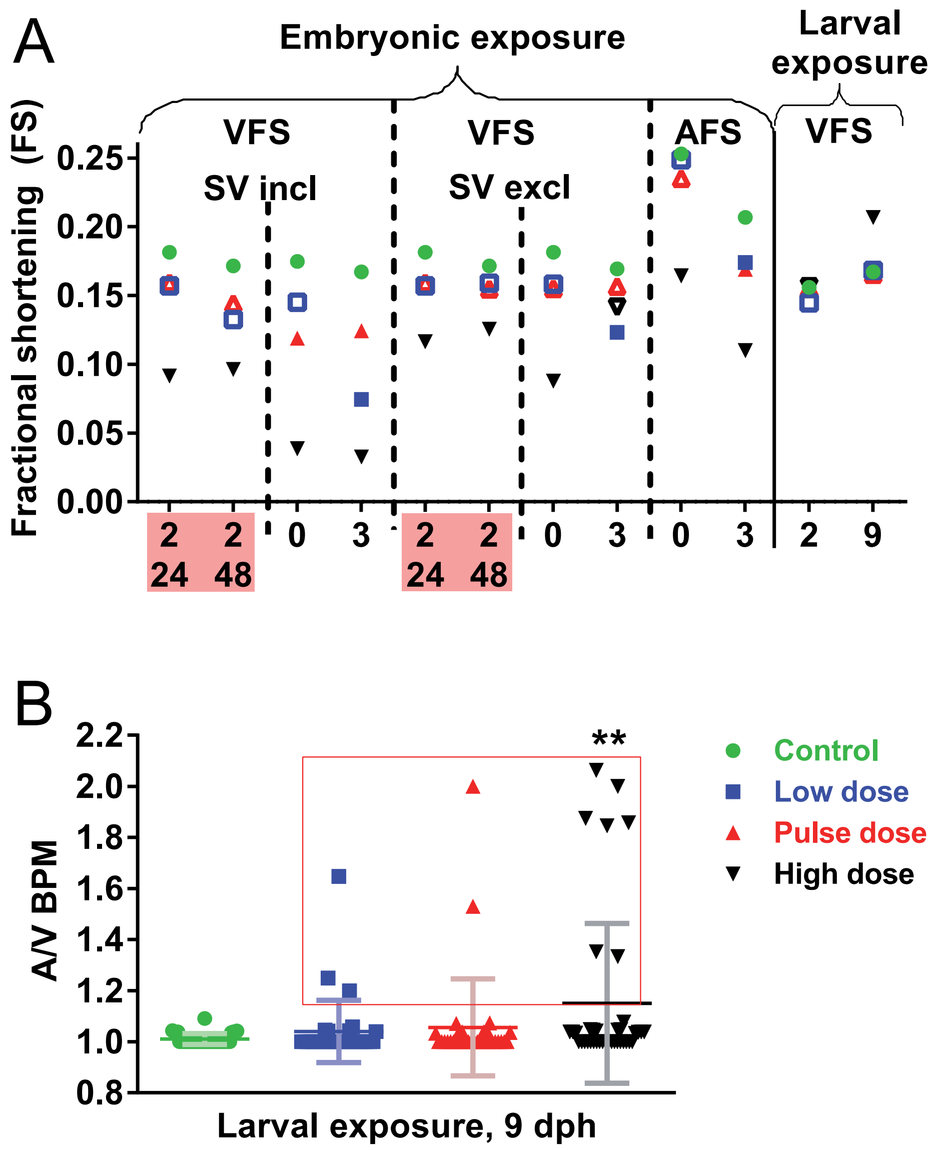

Supplementary Figure S11: Expression of (A) *wnt11*, (B) *cacna1c* and (C) *serca2* in pools during and after embryonic exposure. Significant difference (p =< 0.05) from control is indicated by

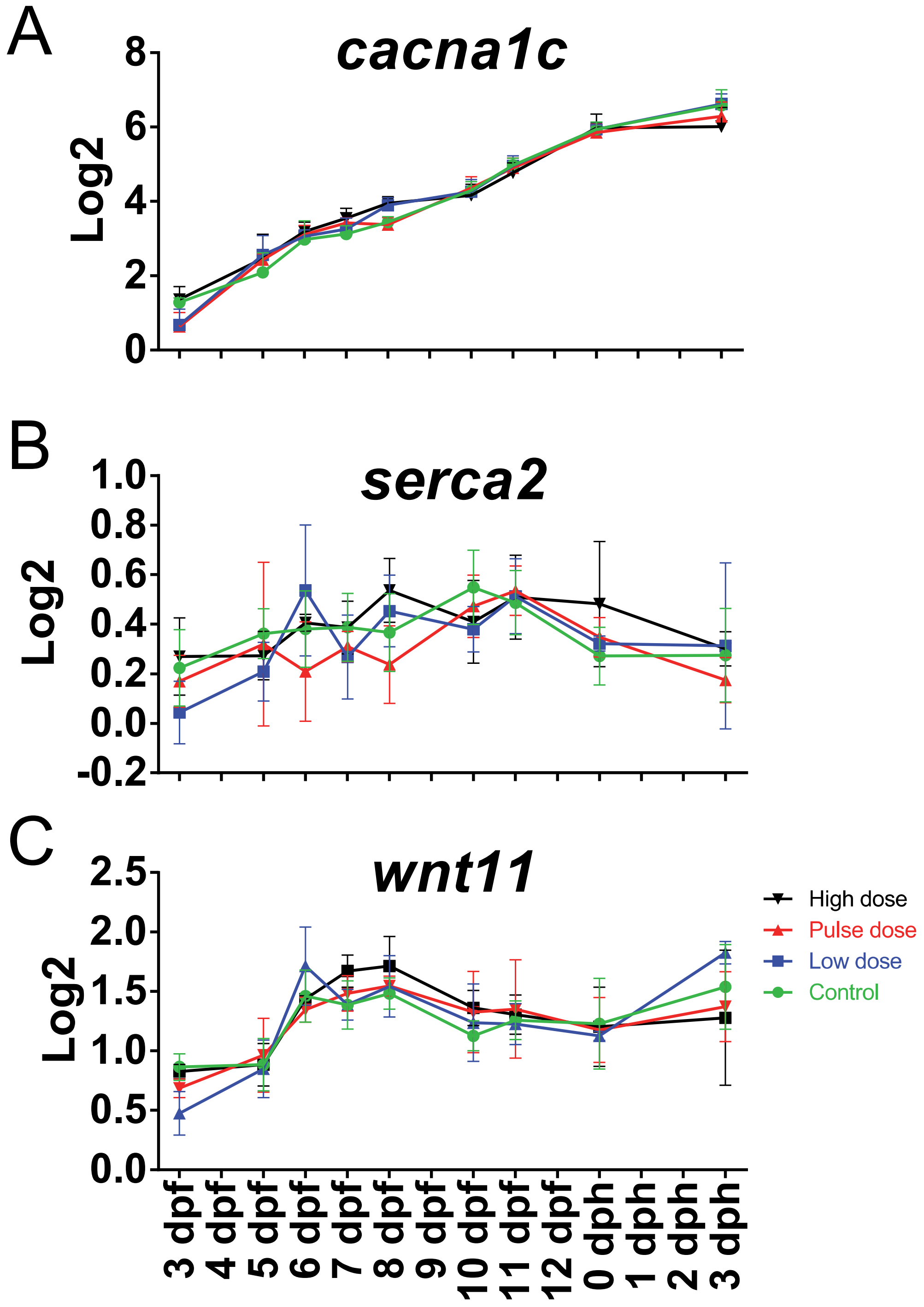

*.

Supplementary Figure S12: Expression of A) *kcnh2*, (B) *ncx1*, (C) *cacna1c*, (D) *serca2* in pools during larval exposure. Significant difference from control is indicated by *(p =< 0.05) and **(p =<0.01).

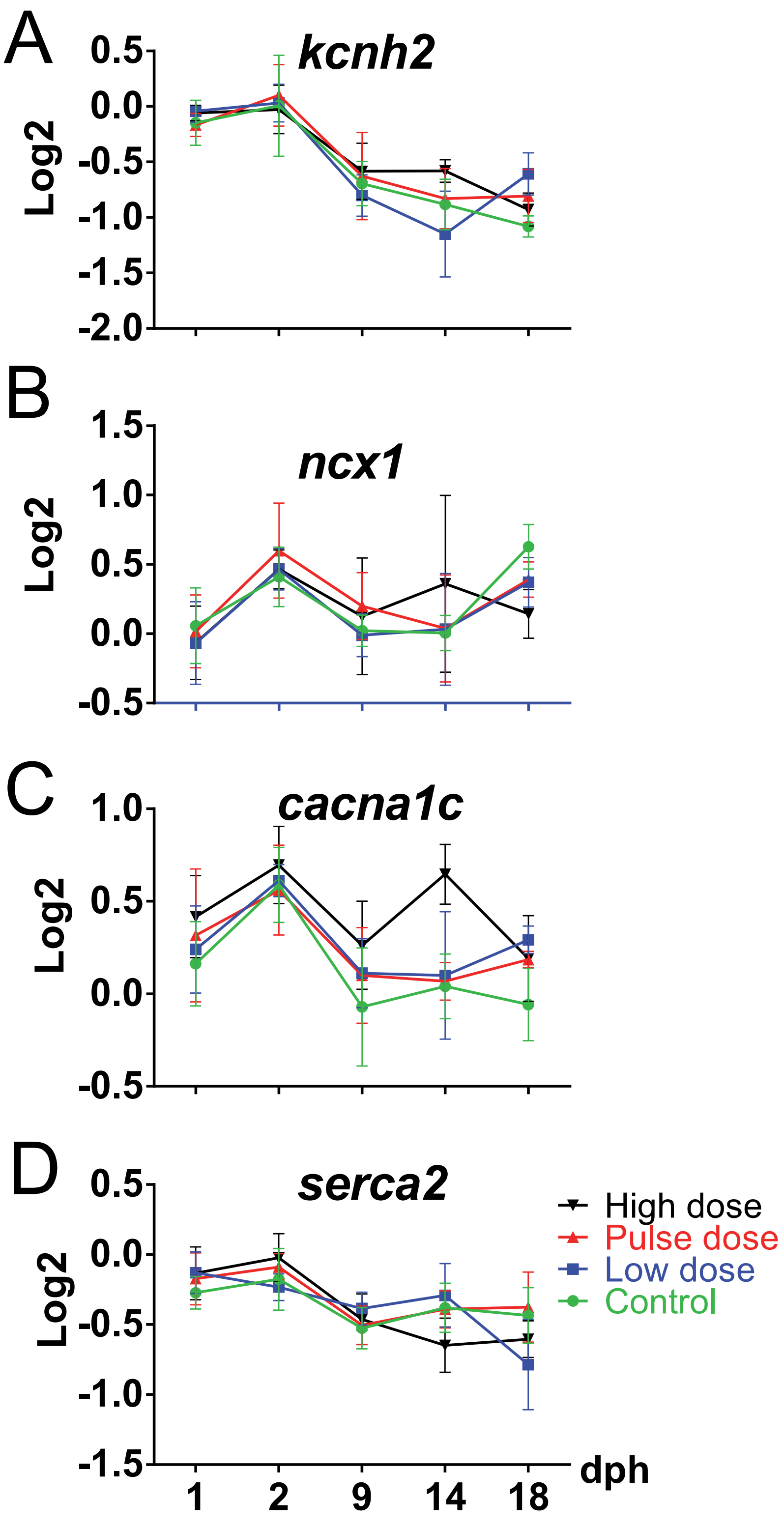

Supplementary Figure S13: Exposure of zebrafish to Heidrun oil. Control and exposed larva after 48 hours (A) and (B) and 72 hours (C) and (D), respectively. Pericardial edema, intracranial haemorrhage poor looping is indicated by black arrow, white arrow and asterisk, respectively. Percentage of edema and intracranial hemorrhage, respectively, in control (E) and exposed (F) animals.

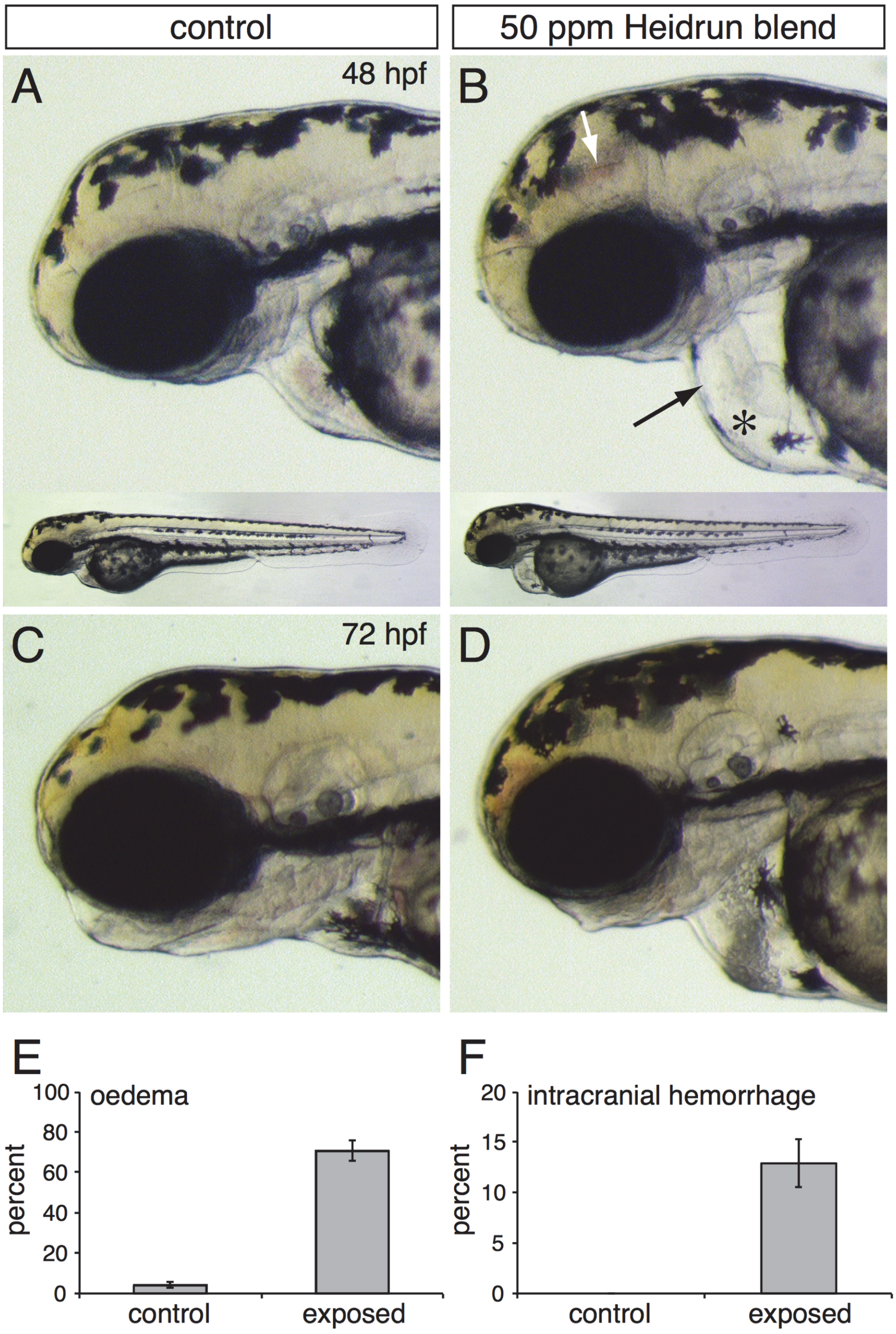

Supplementary Figure S14: Experimental design for the embryonic and larval exposures with sampling points indicated by images of embryos or larvae. (A) Exposure regime for embryonic exposure. Exposure started at 2 dpf and ended at 10 dpf. Video imaging was performed at 9 dpf, 0 dph and 3 dph. Additionally, aliquots of embryos were transferred to tanks with clean seawater after 24 and 48 hours of exposure. These were only sampled and video-imaged at 2 dph. (B) Exposure regime for larval exposure. Exposure started at 0 dph and ended at 18 dph. Larvae were video imaged at 2 dph and 9 dph.

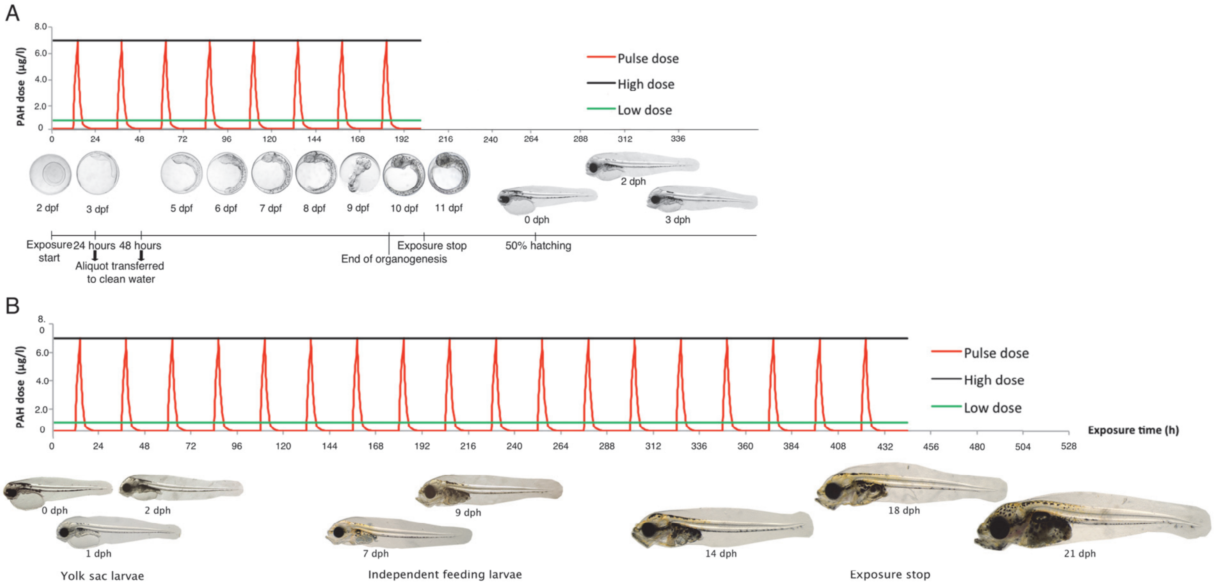

Supplementary Table S1: PAH concentration in water during embryonic exposure (ng/L) (A), in embryos (ng/g) (B), in water during larval exposure (ng/L) (C) and in larvae (ng/g) (D). The following PAH compounds were measured: C0, C1-, C2-, C3-naphthalenes (N), anthracene (ANT), acenaphthylene (AC), acenaphtene (AE), fluorene (F), phenanthrene (P), C1-, C2-, C3-phenanthrene/anthracenes (P/A), C0, C1-, C2-, C3-dibenzothiophenes (D), fluoranthene (FL), pyrene (PY), benzo[a]anthracene (BAA), chrysene (C), benzo[bjk]fluoranthene (BKF), benzo[e]pyrene (BEP), benzo[a]pyrene (BAP), perylene (PER), benzo[ghi]perylene (BZP), indeno[1,2,3-cd]pyrene (IND) and dibenzo[a,h]anthracene (DBA)

A	Control	Low doses	Pulse (low)	Pulse (High)	High
**N**	1 ± 0	23 ± 1	2 ± 0	303 ± 44	282 ± 4
**N1**	3 ± 1	85 ± 5	11 ± 5	1054 ± 139	1005 ± 10
**N2**	3 ± 1	149 ± 13	16 ± 9	1907 ± 296	1859 ± 67
**N3**	8 ± 1	97 ± 6	14 ± 5	762 ± 97	838 ± 21
**N4**	0.7 ± 0.3	3.5 ± 0.3	1.8 ± 0.8	0.2 ± 0.1	0.2 ± 0.0
**AC**	0.1 ± 0.0	0.5 ± 0.4	0.1 ± 0.1	9 ± 2	8.5 ± 0.6
**AE**	0.1 ± 0.0	0.9 ± 0.1	0.1 ± 0.0	11 ± 2	11.3 ± 0.5
**F**	0.35 ± 0.04	8.0 ± 0.9	1.5 ± 0.8	0.4 ± 0.2	0.8 ± 0.6
**D**	0.11 ± 0.01	3.9 ± 0.5	0.6 ± 0.3	45 ± 9	45 ± 2
**D1**	0.26 ± 0.09	13.0 ± 1.5	1.9 ± 0.8	169 ± 32	156 ± 10
**D2**	1 ± 1	33 ± 5	7 ± 0.4	430 ± 82	423 ± 28
**D3**	2.4 ± 0.5	31 ± 4	3 ± 1	444 ± 82	426 ± 31
**P**	0.52 ± 0.04	12 ± 1	2.1 ± 0.9	136 ± 24	136 ± 5
**P1**	0.6 ± 0.2	30 ± 4	5 ± 2	376 ± 73	359 ± 17
**P2**	1 ± 2	44 ± 7	11 ± 5	560 ± 107	552 ± 36
**P3**	1 ± 2	41 ± 6	8 ± 1	563 ± 117	550 ± 31
**ANT**	0.08 ± 0.01	0.29 ± 0.03	0.12 ± 0.03	3.3 ± 0.7	3.0 ± 0.2
**FL**	0.5 ± 0.2	1.13 ± 0.09	0.9 ± 0.6	10 ± 2	11 ± 2
**PY**	1 ± 1	1.5 ± 0.1	3 ± 3	14 ± 2	17 ± 6
**BAA**	0.03 ± 0.01	0.21 ± 0.04	0.09 ± 0.01	3.0 ± 0.8	2.9 ± 0.2
**C**	0.06 ± 0.04	0.7 ± 0.1	0.20 ± 0.07	8 ± 1	7.1 ± 0.5
**C1**	0.01 ± 0.02	0.3 ± 0.2	0.04 ± 0.06	6 ± 1	5.6 ± 0.4
**C2**	0.02 ± 0.02	0.15 ± 0.01	0.04 ± 0.01	2.6 ± 0.4	2.1 ± 0.4
**C3**	0.01 ± 0.02	0.1 ± 0.1	0.01 ± 0.01	0.2 ± 0.3	0.5 ± 0.2
**BBF**	0.08 ± 0.02	0.31 ± 0.03	0.18 ± 0.01	4.1 ± 0.7	4.0 ± 0.5
**BKF**	0.04 ± 0.02	0.09 ± 0.02	0.08 ± 0.01	0.9 ± 0.5	0.8 ± 0.1
**BEP**	0.08 ± 0.02	0.66 ± 0.07	0.18 ± 0.01	7.4 ± 1.5	7.4 ± 0.6
**BAP**	0.05 ± 0.02	0.14 ± 0.01	0.09 ± 0.01	1.9 ± 0.4	2.0 ± 0.2
**PER**	0.01 ± 0.02	0.20 ± 0.04	0.03 ± 0.04	1.8 ± 0.3	2.1 ± 0.2
**IDP**	0.01 ± 0.02	0.02 ± 0.01	0.07 ± 0.02	0.2 ± 0.1	0.3 ± 0.1
**DBA**	0.04 ± 0.03	0.01 ± 0.02	0.08 ± 0.07	0.4 ± 0.1	0.5 ± 0.2
**BGP**	0.04 ± 0.04	0.10 ± 0.05	0.00 ± 0.00	1.6 ± 0.4	1.7 ± 0.5
**∑PAH**	**26 ± 10**	**580 ± 53**	**90 ± 24**	**6834 ± 1033**	**6720 ± 230**

B	Control	Low	Pulse	High
**N**	0.08 ± 0.06	0.61 ± 0.24	0.09 ± 0.15	5 ± 2
**N1**	0.01 ± 0.01	11 ± 1	12 ± 3	86 ± 37
**N2**	4.0 ± 0.4	40 ± 4	49 ± 1	378 ± 152
**N3**	7 ± 2	49 ± 5	58 ± 6	620 ± 256
**N4**	4 ± 2	25 ± 3	33 ± 5	518 ± 236
**AC**	0.00 ± 0.00	0.2 ± 0.1	0.21 ± 0.09	4 ± 2
**AE**	0.00 ± 0.00	0.2 ± 0.2	0.36 ± 0.09	6 ± 3
**F**	0.04 ± 0.07	3.8 ± 0.5	3.4 ± 0.7	51 ± 22
**D**	0.03 ± 0.06	3.0 ± 0.4	2.7 ± 0.5	40 ± 17
**D1**	0.3 ± 0.2	3.1 ± 0.5	3 ± 1	74 ± 35
**D2**	2 ± 1	9 ± 1	11 ± 6	211 ± 100
**D3**	1.6 ± 0.9	7.9 ± 0.2	15 ± 7	163 ± 73
**P**	0.03 ± 0.06	3.6 ± 0.9	2 ± 1	71 ± 32
**P1**	2 ± 1	0.00 ± 0.00	8 ± 4	223 ± 107
**P2**	4 ± 2	11.21 ± 2.35	17 ± 10	311 ± 145
**P3**	2 ± 1	15 ± 1	16 ± 7	125 ± 47
**ANT**	0.00 ± 0.00	7 ± 1	0.00 ± 0.00	0.00 ± 0.00
**FL**	0.25 ± 0.14	0.37 ± 0.09	0.6 ± 0.3	10 ± 4
**PY**	0.11 ± 0.02	0.32 ± 0.05	0.3 ± 0.1	7 ± 3
**BAA**	0.01 ± 0.01	0.04 ± 0.00	0.07 ± 0.06	0.9 ± 0.4
**C**	0.11 ± 0.06	0.38 ± 0.03	0.7 ± 0.4	7 ± 3
**C1**	0.00 ± 0.00	1.14 ± 0.08	2 ± 1	16 ± 6
**C2**	0.00 ± 0.00	1.3 ± 0.2	3 ± 1	13 ± 4
**C3**	0.00 ± 0.00	2.4 ± 0.5	3.8 ± 0.8	13 ± 3
**BBF**	0.03 ± 0.02	0.19 ± 0.03	0.4 ± 0.1	1.7 ± 0.6
**BKF**	0.02 ± 0.01	0.07 ± 0.01	0.14 ± 0.06	0.6 ± 0.3
**BEP**	0.05 ± 0.02	0.24 ± 0.06	0.4 ± 0.2	2.2 ± 0.9
**BAP**	0.00 ± 0.01	0.04 ± 0.02	0.11 ± 0.09	0.5 ± 0.1
**PER**	0.02 ± 0.00	0.05 ± 0.01	0.07 ± 0.03	0.5 ± 0.2
**IDP**	0.02 ± 0.02	0.08 ± 0.03	0.14 ± 0.04	0.4 ± 0.2
**DBA**	0.01 ± 0.01	0.05 ± 0.02	0.11 ± 0.03	0.4 ± 0.2
**BGP**	0.04 ± 0.01	0.24 ± 0.01	0.43 ± 0.06	1.2 ± 0.4
**∑PAH**	**27 ± 10**	**196 ± 18**	**243 ± 50**	**2962 ± 1258**

C	Control	Low doses	Pulse (low)	Pulse (High)	High
**N**	1 ± 0	24 ± 2	2 ± 0	264 ± 14	255 ± 16
**N1**	4 ± 1	88 ± 7	13 ± 12	926 ± 59	972 ± 43
**N2**	6 ± 2	162 ± 16	52 ± 70	1736 ± 144	1941 ± 46
**N3**	10 ± 3	91 ± 8	49 ± 40	737 ± 21	562 ± 56
**N4**	2.6 ± 0.1	0.4 ± 0.3	1.0 ± 0.7	0.2 ± 0.1	0.1 ± 0.1
**AC**	0.1 ± 0.0	0.5 ± 0.4	0.2 ± 0.3	8.1 ± 0.7	8.8 ± 0.3
**AE**	0.1 ± 0.0	0.9 ± 0.1	0.3 ± 0.3	9.5 ± 1.0	11.2 ± 1.0
**F**	0.38 ± 0.06	0.36 ± 0.65	0.31 ± 0.20	0.42 ± 0.18	0.96 ± 0.43
**D**	0.09 ± 0.03	4 ± 0	2 ± 2	38 ± 4	48 ± 2
**D1**	0.15 ± 0.02	15 ± 3	10 ± 9	156 ± 20	212 ± 21
**D2**	0.21 ± 0.09	45 ± 9	30 ± 24	369 ± 50	598 ± 114
**D3**	0.2 ± 0.2	48 ± 8	30 ± 23	393 ± 58	686 ± 162
**P**	0.51 ± 0.05	12 ± 1	6 ± 6	113 ± 9	143 ± 8
**P1**	0.48 ± 0.06	35 ± 7	24 ± 21	346 ± 42	484 ± 71
**P2**	0.3 ± 0.1	59 ± 12	39 ± 30	478 ± 64	760 ± 145
**P3**	0.3 ± 0.3	63 ± 11	38 ± 29	517 ± 62	890 ± 201
**ANT**	0.08 ± 0.01	0.33 ± 0.06	0.3 ± 0.1	2.6 ± 0.6	4.4 ± 0.7
**FL**	0.37 ± 0.04	1.4 ± 0.2	1.1 ± 0.6	9.4 ± 0.7	15 ± 3
**PY**	0.31 ± 0.03	1.86 ± 0.40	1.3 ± 0.8	13 ± 1	21 ± 4
**BAA**	0.07 ± 0.04	0.35 ± 0.02	0.2 ± 0.1	2.4 ± 0.2	4 ± 1
**C**	0.11 ± 0.05	0.93 ± 0.13	0.7 ± 0.3	6.7 ± 0.4	10 ± 3
**C1**	0.06 ± 0.05	0.55 ± 0.08	0.3 ± 0.2	4.4 ± 0.5	2 ± 3
**C2**	0.05 ± 0.05	0.26 ± 0.04	0.1 ± 0.1	2.9 ± 0.3	1 ± 2
**C3**	0.08 ± 0.04	0.05 ± 0.06	0.06 ± 0.05	0.4 ± 0.4	0.3 ± 0.6
**BBF**	0.13 ± 0.07	0.54 ± 0.09	0.3 ± 0.3	3.9 ± 0.2	6.6 ± 1.5
**BKF**	0.09 ± 0.03	0.4 ± 0.2	0.3 ± 0.3	0.9 ± 0.2	1.3 ± 0.5
**BEP**	0.14 ± 0.07	0.9 ± 0.1	0.5 ± 0.4	6.3 ± 0.6	2 ± 4
**BAP**	0.11 ± 0.07	0.24 ± 0.02	0.15 ± 0.08	1.8 ± 0.2	3.0 ± 0.8
**PER**	0.5 ± 0.4	0.5 ± 0.1	0.45 ± 0.07	1.7 ± 0.3	0.8 ± 0.9
**IDP**	0.1 ± 0.1	0.12 ± 0.06	0.05 ± 0.06	0.7 ± 0.6	0.8 ± 0.5
**DBA**	0.09 ± 0.02	0.18 ± 0.03	0.12 ± 0.16	1.0 ± 0.4	1.4 ± 0.9
**BGP**	0.1 ± 0.1	0.24 ± 0.04	0.15 ± 0.13	2.0 ± 0.1	3.2 ± 0.8
**∑PAH**	**29 ± 5**	**656 ± 82**	**301 ± 268**	**6151 ± 474**	**7648 ± 622**

D	Control	Low	Pulse	High
**N**	0.00 ± 0.00	0.9 ± 0.2	0.09 ± 0.05	4 ± 1
**N1**	0.00 ± 0.01	8.0 ± 0.7	1.5 ± 0.5	41 ± 8
**N2**	1.3 ± 0.2	19 ± 2	11 ± 4	133 ± 20
**N3**	1.9 ± 0.3	18 ± 2	17 ± 6	169 ± 29
**N4**	0.8 ± 0.1	7.1 ± 0.7	11 ± 4	116 ± 24
**AC**	0.00 ± 0.00	0.19 ± 0.03	0.2 ± 0.1	1.5 ± 0.3
**AE**	0.00 ± 0.00	0.24 ± 0.08	0.2 ± 0.1	2.1 ± 0.3
**F**	0.00 ± 0.00	1.28 ± 0.08	0.6 ± 0.2	12 ± 3
**D**	0.00 ± 0.00	0.77 ± 0.07	0.5 ± 0.2	10 ± 3
**D1**	0.08 ± 0.01	0.95 ± 0.23	0.9 ± 0.3	14 ± 4
**D2**	0.33 ± 0.07	4 ± 2	5 ± 1	46 ± 18
**D3**	0.00 ± 0.00	6 ± 6	8 ± 3	46 ± 25
**P**	0.00 ± 0.00	0.77 ± 0.09	0.19 ± 0.20	16 ± 5
**P1**	0.00 ± 0.01	2.8 ± 0.5	3 ± 1	48 ± 13
**P2**	0.00 ± 0.00	7 ± 4	10 ± 2	86 ± 37
**P3**	0.00 ± 0.00	4 ± 2	7 ± 3	39 ± 18
**ANT**	0.00 ± 0.00	0.00 ± 0.00	0.00 ± 0.00	0.00 ± 0.00
**FL**	0.02 ± 0.00	0.2 ± 0.2	0.22 ± 0.07	2 ± 1
**PY**	0.01 ± 0.01	0.4 ± 0.5	0.15 ± 0.05	2 ± 1
**BAA**	0.00 ± 0.00	0.02 ± 0.01	0.06 ± 0.02	0.4 ± 0.2
**C**	0.02 ± 0.01	0.2 ± 0.1	0.4 ± 0.1	2.1 ± 0.7
**C1**	0.00 ± 0.00	0.76 ± 0.47	1.2 ± 0.6	6 ± 3
**C2**	0.00 ± 0.00	0.00 ± 0.00	0.00 ± 0.00	4 ± 3
**C3**	0.00 ± 0.00	0.47 ± 0.41	1.3 ± 0.5	4 ± 2
**BBF**	0.00 ± 0.00	0.28 ± 0.40	0.2 ± 0.1	0.6 ± 0.4
**BKF**	0.00 ± 0.00	0.08 ± 0.09	0.1 ± 0.0	0.2 ± 0.1
**BEP**	0.00 ± 0.00	0.40 ± 0.57	0.2 ± 0.1	0.7 ± 0.4
**BAP**	0.00 ± 0.00	0.05 ± 0.06	0.04 ± 0.03	0.2 ± 0.1
**PER**	0.00 ± 0.00	0.01 ± 0.01	0.04 ± 0.03	0.2 ± 0.1
**IDP**	0.00 ± 0.00	0.34 ± 0.59	0.03 ± 0.03	0.10 ± 0.08
**DBA**	0.00 ± 0.00	0.05 ± 0.06	0.04 ± 0.02	0.13 ± 0.06
**BGP**	0.00 ± 0.00	1 ± 2	0.10 ± 0.08	0.3 ± 0.2
**∑PAH**	**5 ± 1**	**86 ± 15**	**81 ± 24**	**808 ± 185**

Supplementary Table S2: Two-way ANOVA table. F-values and p-values for time, dose and interaction for *cyp1a*, *kcnh2*, *ncx1*, *cacna1c* and *serca2*.

Exposure	Gene	Interaction		Time		Dose	
		F-value	p-value	F-value	p-value	F-value	p-value
Embryonic	*cyp1a*	27.23	<0.0001	82.89	<0.0001	693.1	<0.0001
Embryonic	*kcnh2*	2.627	0.0003	46.17	<0.0001	4.880	0.0030
Embryonic	*ncx1*	1.159	0.2946	464.8	<0.0001	3.334	0.0219
Embryonic	*wnt11*	1.173	0.2813	21.81	<0.0001	0.3428	0.7944
Embryonic	*cacna1c*	1.744	0.0274	364.1	<0.0001	0.9192	0.4340
Embryonic	*serca2*	1.212	0.2453	8.204	<0.0001	2.180	0.0939
Larval	*cyp1a*	2.934	0.0012	13.96	<0.0001	543.5	<0.0001
Larval	*kcnh2*	1.541	0.1355	50.21	<0.0001	1.008	0.3959
Larval	*ncx1*	0.8835	0.5681	8.245	<0.0001	0.3303	0.8034
Larval	*wnt11*	1.262	0.2646	7.885	<0.0001	15.87	<0.0001
Larval	*cacna1c*	1.028	0.4370	12.150	<0.0001	5.905	0.0014
Larval	*serca2*	2.120	0.0287	17.67	<0.0001	0.5948	0.6208

Supplementary Table S3: Real time qPCR primer and probe sequences and amplication efficiency, linearity and amplicon length for *cyp1a, wnt11, kcnh2, ncx1, cacna1c, serca2* and *ef1a.* E, efficiency (%); R^2, linearity standard curve; A, amplicon length (bp); Q, quencher.

Gene	Forward primer 5´-3´	Reverse primer 5´-3´	Probe 5´-3´	E	R^2	A	Q
cyp1a	CCTCCTTCCTGCCCTTCAC	TTGGGAATGAAGTAGCCATTGA	6FAM-CCTCACTGCGCCACAAAAGACACATC	110	0.997	83	Tamra
wnt11	AACCCCTACACCGAGAAAC	CTCGACTATCCTTTCGCAC	FAM-TGGTGCTGCTACGTCACCTGCAAGAA	106	0.997	93	Tamra
kcnh2	ACGTGAACCACAACGACGAG	AGCCAGAGCGGAAGATAAGCAG	FAM-<br>TTACATGACCGGGTGGTTCGCCATCGACCTTT	109	0.982	126	Tamra
ncx1	CCGCCCTCATCTACATGTTC	ACGTGATGACCTCGATGGAG	FAM-ATGTCCATCATCGCCGACCGCTTCAT	114	0.997	75	Tamra
cacna1c	ATGAGAACAGCTCCCTCAC	ATGATGACGGCGACAAAC	FAM-<br>GCAGCAATTTTGCCATATTCTACTTTGTCAGCTTC	95	0.984	118	Tamra
serca2	TGTTCCCAGTATGAAGCCC	CACCTTCTCAAACACACCC	FAM-TGCAACGACTCATCGCTGGACTTCAAC	101	0.998	102	Tamra
ef1α	ATCGGCGGTATCGGAACAG	GCTTGAGGACACCGGTCTCA	6FAM-ACCCGTGGGCCGTG	111	0.998	55	None

Supplementary Video S1: Silent ventricle following embryonic oil exposure. Movie shows a control 2 dph larva with normal heart function, followed by a 2 dph larva with non-contracting ventricle. Representative larva is from the high dose for 24-hr transient embryonic exposure.

Supplementary Video S2: Atrioventricular conduction block during larval exposure. Movie shows a control 9 dph larva with normal heart function, followed by a 9 dph larva exposed for the high dose with skipping of ventricular contractions.

Supplementary Video S3: Reduced contractility and poor looping in zebrafish exposed to 50 ppm dispersed crude Heidrun oil. Movie shows a control zebrafish embryo (48 hpf) with normal heart shape and function, followed by an exposed embryo with a poorly looped heart showing normal heart rhythm but reduced contractility.
